# A comparison of recovery benefits associated with participation in SMART Recovery, AA, both SMART and AA, or neither, following a new alcohol use disorder recovery attempt

**DOI:** 10.3389/fpsyt.2026.1886846

**Published:** 2026-10-07

**Authors:** John F. Kelly, Eric Boorman, Jenny B. O’Connor

**Affiliations:** 1 Psychiatry Department, Massachusetts General Hospital, Boston, MA, United States; 2 Psychiatry Department, Harvard Medical School, Boston, MA, United States

**Keywords:** addiction, alcoholics anonymous, groups, longitudinal, mutual help, recovery, self-help, smart

## Abstract

**Background:**

Self-Management and Recovery Training (SMART Recovery or “SMART”) is an internationally known “second-wave” addiction recovery mutual-help organization that has grown over several decades. Despite this, little is known empirically about who uses SMART and how they fare on important alcohol-specific outcomes over time relative to individuals using more established options such as Alcoholics Anonymous (AA), or indeed those who utilize both SMART and AA, or neither. Greater knowledge could deepen understanding of the clinical and public utility of SMART in its own right and inform clinical referral options.

**Methods:**

A naturalistic, longitudinal cohort study (N = 361) of individuals recruited between February 2019 and February 2022 who were initiating a new alcohol use disorder (AUD) recovery attempt and self-selected into one of four groups at study entry: (1) SMART Recovery; (2) AA; (3) SMART + AA; and (4) neither SMART nor AA. Assessments were conducted at intake, 3 months, and 6 months and measured a variety of alcohol/drug-related and functioning outcomes.

**Results:**

Compared to the AA and Both groups, SMART Recovery pathway participants exhibited a pattern of less severe clinical pathology and legal system involvement histories, along with higher psychosocial stability and recovery capital. In terms of meeting attendance frequency, the SMART group did not attend as many meetings as the AA or Both groups, but nevertheless was able to achieve levels of improvement in alcohol indicators (i.e., fewer drinking days, fewer heavy drinking days, and fewer alcohol-related consequences) and rates of early AUD remission similar to those of the AA and Both groups.

**Conclusions:**

SMART Recovery may provide a valuable alternative source of recovery-specific support that may be a good fit, particularly for certain individuals with AUD. As such, it serves as a valuable addition to the menu of available community-based recovery-support service options.

## 1 Introduction

Recovery from alcohol and other drug (AOD) disorders is a demanding process often characterized by numerous recovery attempts before full remission is achieved (1, 2). In making these attempts, many seek the help of a variety of resources, such as formal treatment services and community-based recovery support services, including recovery housing (3), recovery coaching (4), and recovery support in educational and work settings (5, 6). By far the most commonly sought sources of help, however, are peer-led mutual-help organizations (MHOs), such as Alcoholics Anonymous (AA) and other 12-step fellowships (7, 8). Newer entities, sometimes referred to as “second-wave” MHOs, also have emerged and grown (9, 10). One of the most well-known of these non-12-step alternatives is Self-Management and Recovery Training (SMART; often referred to as “SMART Recovery”) (11).

SMART differs from the more common 12-step organizations in its philosophy, format, focus, and goals. Whereas 12-step approaches typically follow a purely peer-led model (i.e., all participants have lived experience of addiction and recovery from it) without formal training for meeting facilitators and recommend close-knit relationship ties through “sponsorship” (where an established member with longer-term sobriety—especially early on—typically provides frequent and intensive support and encouragement, role-modeling, and help to newcomers to help them understand and work through the 12-step program; [AA, 2025]), SMART Recovery operates its group meetings through formally trained facilitators, who may or may not be peers themselves (i.e., do not have to have lived experience of addiction and recovery from it) and does not provide for or explicitly recommend such formal social arrangements between its members (11). In addition, while AA has been shown to facilitate recovery by facilitating adaptive social network changes (12), boosting cognitive and behavioral coping skills, recovery self-efficacy, and recovery motivation, and reducing impulsivity and craving (13–22), it is perhaps most well-known for having an explicit spiritual emphasis, which has also been shown to be a salutary mechanism for many in preventing relapse (23–25). In contrast, SMART Recovery is explicitly non-spiritual, and although mechanisms of SMART Recovery have not been formally tested, its approach is founded on more clinically and professionally derived motivational and cognitive-behavioral psychological principles and is, therefore, assumed to convey relapse prevention benefits through those elements. AA is also designed for people who have found they cannot control their use of alcohol despite repeated attempts to do so and thus want to stop completely (26). SMART, in contrast, caters to any substance (alcohol or other drugs) or behavioral addiction problems and allows for goals of reduced use or complete cessation (11, 27).

These resources thus present different “flavors” of recovery support that seem to appeal to different AUD phenotypes (28). Specifically, in some of our prior research, we have found that individuals drawn to SMART tend to have lower levels of clinical pathology, less prior treatment, more limited legal histories, greater psychosocial stability, and more recovery capital than individuals drawn to AA (28). Notably, while these may represent distinct choices regarding recovery support, some individuals attend both (29) and are able to accommodate and utilize these different philosophies and formats. Of note, some individuals also choose neither of these options, preferring instead to find their own “self-managed” path without the use of these commonly used services.

As noted above, our prior work in this area has found that these supportive resources appeal to different people, but has not reported on participation patterns or how individuals fare on outcomes important to recovery while using these resources. Other work (10, 30) has found that when individuals self-select into different recovery support options (including SMART and/or AA), they show similar levels of benefit, especially when their different alcohol use goals are taken into account (30). Adding to this limited empirical literature, the current investigation follows individuals for six months after they start a new AUD recovery attempt and self-select into one of four recovery pathways (i.e., AA, SMART, SMART + AA, Neither AA nor SMART) and examines patterns of AA/SMART meeting participation and the relation between pathway choice and a variety of alcohol/drug outcomes. More specifically, in this paper, we address three main research questions: (1) How similar or different are the four recovery pathway groups at baseline in terms of demographic, clinical, treatment, and legal history characteristics? (2) What are the MHO participation rates and alcohol-related outcomes across these pathway choice groups over 6 months after starting an AUD recovery attempt? (3) What are the effects of pathway group choice on alcohol-related outcomes after controlling for confounding variables? We focus on four main alcohol-related outcomes: percent days drinking (PDD), percent heavy drinking days (PHDD), percent in AUD remission, and alcohol-related consequences.

## 2 Methods

### 2.1 Study overview

This study is a naturalistic, prospective, longitudinal cohort study of 361 individuals making a new recovery attempt from AUD who self-selected into one of four recovery pathways: 1. AA only (“AA”); 2. SMART only (“SMART”); 3. Both SMART + AA (“Both”); and 4. Neither SMART nor AA (“Neither”). Participants underwent assessments at baseline and at 3- and 6-month follow-ups. Assessments included self-report and staff-administered measures conducted via phone and/or Zoom. Baseline visits were conducted from February 2019 to February 2022. The study was fully reviewed and approved by the Institutional Review Board at Mass General Brigham, Boston, MA, USA. For additional details on this study, see (28, 31).

### 2.2 Recruitment

Participants were recruited through SMART Recovery meetings (about 71% of SMART participants), inpatient and outpatient treatment programs, and a variety of commercial recruitment sources during the recruitment period (January 2019 to January 2022). Flyers and postcards for the study were distributed at Massachusetts General Hospital (MGH). SMART facilitators advertised the study at SMART meetings and were provided with recruitment postcards and flyers. The study was also advertised on the SMART website. Additional recruitment methods included ResearchMatch, Partners eCare Research Core (PeRC), TrialFacts, Rally for Recruitment, the Metro Newspaper, radio advertisements, Massachusetts Bay Transportation Authority (MBTA), Facebook, Craigslist, and Reddit. For radio, MBTA, Facebook, and Craigslist advertisements, interested individuals called the study-specific phone line, emailed the study-specific email address, or filled out an online screening form. Individuals were then able to participate in a brief 10–15-minute phone screen, during which eligibility criteria were confirmed. If the individual was eligible to participate, the baseline visit was scheduled, and contact information for two locator contacts who could assist research staff in locating participants was collected.

### 2.3 Inclusion criteria

Participants were required to be 18 years or older, live in the New England or San Diego metropolitan area, and be willing to travel to Boston, Massachusetts, to complete study visits (for New England residents) or to complete study visits remotely (due to COVID-19 and for the San Diego participants). The geographical catchment area eligibility criteria were expanded to include people from the San Diego area in December 2020 to increase the number of SMART participants in the study. Since all visits were conducted remotely beginning in March 2020 due to the COVID-19 pandemic, participants from the New England area were also considered eligible even if they could not travel to the Boston office for assessments. Participants could be using other drugs but had to report alcohol as their primary substance of concern; they were also required to have a self-perceived alcohol problem; to meet current criteria for DSM-5 alcohol use disorder (AUD) using a semi-structured interview; and to have consumed alcohol in the past 90 days and report being currently engaged in a new recovery attempt, defined as “a serious effort to abstain from drinking or to drink without problems in the past 90 days or planning to make one in the next 14 days.” Additionally, participants were required to provide locator contact information for two close friends/family members in case we were unable to contact the participant directly; provide their Social Security number for reimbursement or be willing not to receive reimbursement; provide a urine sample and complete a breathalyzer test (for in-person visits) or a remote saliva test (for remote visits) for biochemical verification; and provide a stable home address and contact information. Bioassay assessments were intended to confirm self-reported alcohol/drug use. These initial bioassay requirements were discontinued following the start of COVID-19 lockdowns, which began in March 2020, and there were insufficient bioassay data of adequate quality to use in analyses.

### 2.4 Procedure

All assessments were initially conducted (before COVID-19) in person with a study research coordinator at our downtown Boston offices at the MGH Recovery Research Institute. Each assessment consisted of staff-administered and self-administered surveys, which were completed via Research Electronic Data Capture (REDCap) (a secure, web-based application designed to support data capture for research studies), a computerized task to assess impulsivity (Go/No-Go task), and biochemical verification tests of abstinence (breathalyzer, urine) for all participants at all time points. For in-person visits, the baseline and follow-up assessments lasted approximately 3 hours. As noted above, due to COVID-19, all assessments were conducted remotely beginning in March 2020. During remote visits, the computerized impulsivity task and urine and breath biochemical verification tests of abstinence were not completed. A web-based version of the computerized task was tested, but the effects of internet speed on results made data unreliable. In lieu of the urine and breathalyzer tests, saliva tests were implemented for remote visits from March 2021 to May 2021 but were discontinued due to documented inconsistent results. Relative to in-person assessments, remote assessments were shorter, with assessments lasting approximately 1.5 hours (for baseline) or 45 minutes (for follow-ups) on the phone and approximately 1 hour for participants to complete surveys individually.

All participants (in-person and remote) agreed to provide their phone numbers and email information and that of two locator contacts so that the participants could be contacted for follow-up assessment reminders. Research staff contacted and confirmed the contact information of the locator contacts as needed if research staff lost touch with the participant. Participants indicated their preferred method of contact (phone call, email, or text message) for receiving automated reminders throughout the project period. In keeping with a validated research follow-up protocol for maximizing retention in clinical addiction research, after the baseline assessment, research staff proactively reached out to participants for reminders and to check if there were any changes to their contact information. Check-ins occurred 1 month, 14 days, 7 days, and 24 hours before the next scheduled visit. These messages were automated and sent using Twilio, a Mass General Brigham-approved REDCap module.

Participants were compensated $45 for the baseline visit and $55 and $60 for the 3- and 6-month follow-up visits, respectively. Payment for each time point was broken into payments for the staff-administered surveys, self-administered surveys, and travel reimbursement. During remote visits due to COVID-19, all participants were still paid the travel reimbursement to maintain the same payment structure used for in-person assessments.

### 2.5 Participants

Study participants’ (N = 361) baseline characteristics and changes over time in outcome variables are described in **Table 1**. Of the 361 individuals assessed and included at baseline, 88% and 85% were reassessed at the 3- and 6-month follow-ups, respectively.

**Table 1:** Demographic, clinical, and legal comparisons between groups (SMART, AA, Both, Neither).

n	Whole sample	SMART	AA	Both	Neither	F / χ^2^ p
	361	76	72	51	162	
Demographic						
Age (M, SD)	46.10 (12.90)	49.01 (13.06)	46.11 (11.57)	45.34 (13.38)	44.97 (13.13)	1.77
Gender (% Female)	52.63 (190)	39.47 (30)^A^	50.00 (36)	52.94 (27)	59.88 (97)^A^	10.57*
Sexual orientation (% LGBT)	21.61 (78)	19.74 (15)	26.39 (19)	21.57 (11)	20.37 (33)	1.15
Hispanic (% yes)	9.14 (33)	15.79 (12) ^A^	1.39 (1) ^A^	9.80 (5)	9.26 (15)	9.56*
Race						18.40**
White	73.96 (267)	88.16 (67) ^ABC^	76.39 (55) ^A^	68.63 (35)^B^	67.90 (110)^C^	
Black or African American	13.85 (50)	1.32 (1)	18.06 (13)	17.65 (9)	16.67 (27)	
Other / Decline to Answer	12.19 (44)	10.53 (8)	5.56 (4)	13.73 (7)	15.43 (25)	
Education						20.96**
High School or Less	12.47 (45)	5.26 (4)	15.28 (11)	15.69 (8)	13.58 (22)	
Some College or Other Degree	35.18 (127)	22.37 (17)	45.83 (33)	29.41 (15)	38.27 (62)	
BA or Higher	50.14 (181)	71.05 (54) ^AB^	37.50 (27)^A^	54.90 (28)	44.44 (72)^B^	
Income						38.34***
Less than $10,000	12.19 (44)	3.95 (3)	16.67 (12)	17.65 (9)	12.35 (20)	
$10,000 to $49,999	40.72 (147)	22.37 (17)	56.94 (41)	49.02 (25)	39.51 (64)	
$50,000 or more	44.88 (162)	72.37 (55)^ABC^	26.39 (19)^AD^	31.37 (16)^B^	44.44 (72)^CD^	
Employed						11.32m
Unemployed	37.12 (134)	27.63 (21)	41.67 (30)	47.06 (24)	36.42 (59)	
Part time / Occasional	18.84 (68)	15.79 (12)	25.00 (18)	17.65 (9)	17.90 (29)	
Full time work	41.83 (151)	55.26 (42)^A^	31.94 (23)^A^	33.33 (17)	42.59 (69)	
Marital status						20.03*
Married	35.46 (128)	52.63 (40)^ABC^	26.39 (19)^A^	29.41 (15)^B^	33.33 (54)^C^	
In a Relationship	10.25 (37)	6.58 (5)	9.72 (7)	7.84 (4)	12.96 (21)	
Single	36.29 (131)	21.05 (16)	47.22 (34)	45.10 (23)	35.80 (58)	
Separated / Divorced / Widowed	15.24 (55)	18.42 (14)	15.28 (11)	15.69 (8)	13.58 (22)	
Location (% from San Diego)	18.01 (65)	38.16 (29)^AB^	5.56 (4)^AC^	39.22 (20)^CD^	7.41 (12)^BD^	56.03***
Clinical						
Alcohol Symptoms (Staff-Admin)	7.61 (3.01)	7.38 (2.93)^AB^	8.77 (2.95)^AC^	8.79 (2.71)^BD^	6.88 (2.93)^CD^	9.21***
% in Remission	6.09 (22)	7.89 (6)	6.94 (5)	7.84 (4)	4.32 (7)	2.06
Psychiatric Distress	14.45 (5.07)	14.63 (4.83)	13.80 (4.50)	14.90 (4.38)	14.50 (5.62)	0.55
Lifetime Mental Health Diagnoses (% Yes)	70.91 (256)	71.05 (54)	76.39 (55)	78.43 (40)	66.05 (107)	4.61
Number of Mental Health Diagnoses	2.05 (2.03)	1.79 (1.74)	2.54 (2.39)^A^	2.34 (2.01)	1.87 (1.96)^A^	2.63*
Prior treatment						
Lifetime Outpatient Treatment	1.76 (2.95)	1.12 (2.03)^AB^	3.35 (3.83)^AC^	2.80 (2.92)^BD^	1.02 (2.48)^CD^	15.37***
Past 90 Day Outpatient Treatment	0.30 (0.82)	0.33 (0.76)	0.53 (1.11)^A^	0.47 (0.78)^B^	0.14 (0.66)^AB^	4.80**
Lifetime Detox	1.84 (3.20)	1.03 (2.09)^AB^	4.06 (4.08)^ACD^	2.92 (3.65)^BD^	0.90 (2.38)^C^	23.46***
Past 90 Day Detox	0.20 (0.52)	0.21 (0.44)^AB^	0.36 (0.61)^C^	0.53 (0.90)^AD^	0.02 (0.14)^BCD^	17.87***
Lifetime Inpatient Treatment	1.46 (2.60)	0.92 (1.87)^AB^	2.85 (3.32)^AC^	2.53 (2.95)^BD^	0.77 (2.02)^CD^	16.52***
Past 90 Day Inpatient Treatment	0.16 (0.45)	0.16 (0.37)^AB^	0.29 (0.54)^C^	0.43 (0.78)^AD^	0.02 (0.14)^BCD^	15.23***
% SUD/MH Service use in Past 90 Days	60.94 (220)	76.32 (58)^A^	73.61 (53)^B^	82.35 (42)^C^	41.36 (67)^ABC^	47.77***
Legal system involvement						
Active Criminal Justice Involvement	9.14 (33)	10.53 (8)	12.50 (9)	11.76 (6)	6.17 (10)	2.88
Lifetime Arrests (% Yes)	54.02 (195)	47.37 (36)^A^	70.83 (51)^AB^	66.67 (34)^C^	45.68 (74)^BC^	14.89**
Number of Arrests in Lifetime (Total)	2.09 (3.09)	1.40 (2.35)^A^	3.08 (3.52)^AB^	2.53 (3.43)	1.82 (2.97)^B^	4.57**

m p <.10, * p <.05, ** p <.01, *** p <.001.

Letters reflect significant differences between groups that have the same superscripted letter.

### 2.6 Measures

Detailed descriptions of all measures are available in Supplement 1. Staff-administered measures assess the following: substance use history including capture of primary outcomes (percent heavy drinking days; percent days abstinent from alcohol/other drugs; Timeline Follow-Back), AUD and substance use disorder (SUD) status and severity (including remission status; Diagnostic Assessment Registration Table [DART]), tobacco use, treatment utilization for physical health problems and alcohol/drug use problems, anti-craving and anti-relapse medications (alcohol and opioids), mental and emotional health diagnoses, hospitalizations, treatment history, psychiatric medication use, social networks, 12-step/MHO attendance history, online resource utilization, SMART involvement, 12-step MHO involvement (MM-HAS), recovery/abstinence time, recovery support services and formal treatment program utilization, substance use change over the past year (YES), impulsivity (Go/No-Go cognitive task), and biochemical verification of substance use (breathalyzer, urine drug screen).

Self-administered measures assess the following: demographics, criminal justice involvement, religiosity and spirituality (RBBS, religious and spiritual intensity, DSES), stress and psychiatric distress (PSS-4, K6), coping (CSS), self-efficacy (A-DSES-20, single item self-efficacy), alcohol/other drug craving (PADCS-5), commitment to sobriety (CSS-5), substance use consequences (SIP-2R), recovery status (questions about recovery, drinking goal), recovery capital (BARC-10), behavioral addictions, medical marijuana use, medication attitudes, impulsive behavior (SUPPS-S), quality of life and psychosocial functioning (TPS, Q-LES-Q, EQ5D3L, EUROHIS-QOL, self-esteem, happiness, and satisfaction with life), and physical health (PSQI, pain VAS, IPAQ, meals).

All measures were administered at each time point except for the Year End Summary (YES), SUD DART, and Questions about Recovery, which were administered at baseline and at 12 and 24 months.

### 2.7 Data analysis plan

#### 2.7.1 Control variables and descriptive statistics

##### 2.7.1.1 Control variables

First, we screened all variables to identify those that predicted study dropout at 3 months or 6 months and then screened baseline variables that predicted each outcome of interest (e.g., % days drinking) so that they could be controlled for in the predictive models (e.g., in testing the main effect of group and the interaction effect of group × time in predicting outcomes). Specifically, we conducted three sets of analyses assessing (1) predictors of study dropout; (2) predictors of the specific outcome variable examined in each model among those variables that differed between the four groups at baseline; and (3) other baseline predictor variables that predicted the specific model outcome variable.

We screened for these predictors first using univariate analyses and included any significant univariate predictors in a combined multivariate model, retaining those variables that independently predicted dropout or the relevant outcome at a p <.10 type I error rate in the multivariate model. A more inclusive p <.10 is consistent with prior research to allow for the maximum possible number of control variables and, by extension, the most conservative estimates of main and interaction effects. Among this final set of control variables, we assessed potential multicollinearity by examining the intercorrelations among them. Specifically, we ran correlations between significant model predictors at each step and investigated any correlation above.70—a more conservative threshold for multicollinearity. Any correlations >.70 among predictor variables were examined, and the predictor with the highest absolute correlation with the specific outcome variable was retained in analyses, with the second redundant predictor removed to reduce multicollinearity issues (32, 33).

##### 2.7.1.2 Descriptive and preliminary inferential statistics

We computed descriptive statistics for the demographic variables across the entire sample and by group (AA, SMART, Both, Neither; **Table 1**) and computed summaries of central tendency and spread for the outcome variables of interest by group and across time (**Table 2**). We also tested for any between-group baseline differences on these variables.

**Table 2:** Table of descriptive statistics of alcohol and drug outcomes and meeting attendance over time.

Follow up	SMART M (SD)	AA M (SD)	Both SMART & AA M (SD)	Neither SMART nor AA M (SD)
Alcohol and drug outcomes				
PDD				
0M	44.58 (31.34)	38.69 (30.66)	27.22 (21.64)	54.09 (32.13)
3M	23.81 (30.94)	20.60 (29.79)	19.35 (27.71)	39.89 (36.91)
6M	23.21 (30.57)	28.75 (33.71)	21.93 (28.94)	32.55 (33.83)
% Heavy drinking days				
0M	32.13 (29.15)	32.91 (28.66)	22.69 (20.57)	36.11 (35.15)
3M	12.79 (20.55)	15.22 (24.77)	13.65 (23.97)	18.45 (29.72)
6M	13.19 (24.00)	22.13 (29.16)	14.68 (25.53)	15.50 (26.73)
% in AUD remission (% N)				
0M	7.89 (6)	6.94 (5)	7.84 (4)	4.32 (7)
3M	47.37 (36)	40.28 (29)	41.18 (21)	27.16 (44)
6M	42.11 (32)	36.11 (26)	45.10 (23)	40.12 (65)
Consequences				
0M	21.11 (10.39)	24.41 (12.22)	25.29 (11.31)	18.97 (11.00)
3M	10.03 (10.80)	12.77 (13.95)	10.88 (12.35)	13.64 (11.19)
6M	8.37 (10.54)	13.97 (14.27)	9.95 (11.33)	10.94 (10.92)
Mutual help involvement				
Total AA days				
0M	0.09 (0.33)	22.69 (26.44)	19.96 (23.28)	0.18 (1.99)
3M	0.44 (2.23)	23.85 (31.70)	18.34 (28.91)	0.34 (2.37)
6M	0.23 (1.21)	26.25 (34.02)	18.53 (29.02)	2.27 (10.52)
Total SMART days				
0M	9.50 (14.87)	0.00 (0.00)	11.06 (15.28)	0.00 (0.00)
3M	13.73 (17.28)	0.20 (0.81)	9.87 (12.94)	0.03 (0.34)
6M	8.50 (12.53)	0.00 (0.00)	7.89 (12.12)	0.09 (0.81)
Total MHO days				
0M	9.59 (14.92)	22.69 (26.44)	31.02 (31.27)	0.18 (1.99)
3M	14.16 (17.13)	24.05 (31.71)	28.21 (34.01)	0.37 (2.39)
6M	8.73 (12.54)	26.25 (34.02)	26.42 (32.32)	2.36 (10.60)

##### 2.7.1.3 Recoding outcomes

Given that percent days drinking (PDD) and percent days heavy drinking (PHDD; a heavy drinking day was defined as 4 or more standard U.S. drinks in a day for men and 3 or more for women) displayed severe deviations from normality (negative skew), distorting the accuracy of the parameter effect estimates, after examining the frequency score distribution, we decided to transform both variables to reflect the number of drinking days/heavy drinking days per week. Although linear transformations (e.g., square root, log, etc.; 24) have been used to reduce skewness and kurtosis, resulting model coefficients can be difficult to interpret. Consequently, in order to obtain more intuitive and clinically useful findings, we elected to recode variables reflecting drinking days/heavy drinking days at an average weekly level. Percent days drinking was recoded into a four-category ordinal variable reflecting frequency of alcohol use where 1 = zero drinking days per week; 2 = 1 or more, but less than 2, drinking days per week, on average; 3 = 2 or more, but less than 4, drinking days per week, on average; and 4 = 4 or more drinking days per week, on average. Similarly, PHDD was recoded to a four-category ordinal variable reflecting 1 = zero heavy drinking days per week; 2 = 1 or more but less than 2 heavy drinking days per week, on average; 3 = 2 or more but less than 4 heavy drinking days per week, on average; and 4 = 4 or more heavy drinking days per week, on average.

With respect to consequences (i.e., the SIP-2R instrument, which examined consequences during each of the past 90 days using items such as “Because of my drinking or drug use (in the past 90 days) I have not eaten properly”; “When drinking or using drugs (in the past 90 days) I have done impulsive things that I have regretted later.”), we observed that a substantial proportion of individuals (3-month n = 50, 6-month n = 46) reported experiencing consequences from alcohol use even though the questionnaire clearly refers to the past 3 months at each time point (i.e., “During the past 3 months how often has this happened to you?”), and despite the fact that these individuals reported no alcohol use in the past 3 months. We believe these individuals misinterpreted the timeframe pertaining to the occurrence of these consequences (e.g., several people reported an alcohol-related accident in the past 3 months despite reporting zero alcohol use). Thus, we recoded these individuals to reflect 0 consequences, as they reported zero alcohol use and therefore could not experience alcohol-related consequences, consistent with prior research (10). Subsequent research may seek to further evaluate the SIP to determine the extent to which this measure is capturing both present and residual lifetime consequences. We ran analyses with and without these cases included and found a similar but more accentuated pattern of results when these individuals with reported consequences were included.

#### 2.7.2 Main effects of group and interactions of group × time on outcome variables

To test the effect of group membership (e.g., AA, SMART, Both, Neither) on the outcomes, we first ran unadjusted models that did not include any control variables (Model 0), followed by different incremental adjusted models that controlled for (a) any baseline predictors of dropout (Model 1); (b) the variables in (a) plus any predictors of the outcome variable from the variables that differed between groups at baseline (Model 2); and (c) the variables in (a) and (b) plus any other baseline predictors of the outcome variable (Model 3). This incremental modeling approach, which has been consistently used in prior research (e.g., Kelly et al., 2013; Bergman et al., 2026), was used to uncover the influence that any of the different types of control variables may have had on the relationship between the main variable of interest (group membership) and each outcome variable (e.g., PDD, % days heavy drinking, etc.).

## 3 Results

### 3.1 How similar or different are the four recovery pathway groups at baseline in terms of demographic, clinical, treatment, and legal history characteristics?

Group differences at baseline have been reported elsewhere in detail (28) but are also described here. At baseline, the four groups differed on several demographic, clinical, treatment history, and legal history variables. Specifically, as shown in **Table 1**, and similar to what was reported previously (28), we observed statistically significant group differences in several demographic variables (i.e., gender, ethnicity, race, education, income, employment, marital status, and recruitment location); clinical and treatment variables (i.e., AUD symptoms, lifetime number of mental health diagnoses, lifetime outpatient treatment, past 90-day outpatient treatment, lifetime detox, past 90-day detox treatment, lifetime inpatient treatment, past 90-day inpatient treatment, and % SUD/mental health (MH) service use in the past 90 days); and legal system involvement variables (i.e., % of lifetime arrests and number of arrests in lifetime).

To determine the direction of effects, we conducted *post hoc* analyses. For continuous outcomes (e.g., AUD symptoms, lifetime number of mental health diagnoses, and lifetime outpatient treatment), we used Tukey’s honestly significant difference (HSD) test to account for multiple comparisons across groups; for categorical variables (e.g., gender, ethnicity, and race), we used *post hoc* pairwise comparisons using the Holm correction.

#### 3.1.1 Demographic differences between groups at baseline

With respect to demographic variables, we observed significant differences in the proportion of female participants, whereby the SMART group had a lower percentage of female participants (40%) compared to the Neither group (60%). We observed significant differences in ethnicity between the SMART and AA groups whereby the SMART group had a higher proportion of Hispanic participants (16%) compared to the AA group (1%; this may have been due to the fact that a large number of SMART participants were recruited in San Diego, CA, where the Hispanic population is much larger). We observed significant differences in race whereby the SMART group had a higher percentage of individuals identifying as White (88%) compared to the AA group, Both group, and Neither group (76%, 69%, and 68%, respectively). Large and significant differences were also observed in education, whereby the SMART group had a higher percentage of participants with a bachelor’s degree or higher (71%) compared to the AA group and the Neither group (38% and 44%, respectively). Large and significant differences in income were also observed whereby the SMART group had a much higher percentage of individuals making $50K or more (72%) compared to the AA group, the Both group, or the Neither group (26%, 31%, and 44%, respectively). In addition, the AA group had a lower percentage of individuals making $50K or more (26%) compared to the Neither group (44%). With respect to marital status, the SMART group had a much higher percentage of individuals who were married (53%) compared to the AA group, the Both group, or the Neither group (26%, 29%, and 33%, respectively), and regarding employment, the SMART group had a higher percentage of individuals who were employed full-time (55%) compared to the AA group (32%). As noted above regarding Hispanic group composition, the SMART group had a much higher percentage of the sample recruited from San Diego (38%) compared to the AA group and the Neither group (6% and 7%, respectively). Likewise, the Both group had a higher percentage of the sample recruited from San Diego (39%) compared to the AA group and the Neither group (6% and 7%, respectively).

#### 3.1.2 Clinical and prior treatment variable differences between groups at baseline

With respect to baseline clinical variables, for AUD symptoms, the SMART group had fewer AUD symptoms on average (M = 7.38) compared to the AA group (M = 8.77) and the Both group. Likewise, the Neither group had fewer AUD symptoms on average (M = 6.88) compared to the AA group and the Both group (Ms = 8.77, 8.79, respectively). With respect to the number of mental health diagnoses, the AA group had more mental health diagnoses on average (M = 2.54) compared to the Neither group (M = 1.87).

With respect to prior formal treatment, for lifetime outpatient treatment, the SMART group had a lower frequency of treatment on average (M = 1.12) than the AA group or the Both group (Ms = 3.35, 2.80, respectively). Likewise, the Neither group had fewer treatments on average (M = 1.02) than the AA group or the Both group (Ms = 3.35, 2.80). With respect to past 90-day outpatient treatments, the Neither group had fewer treatments on average (M = 0.14) compared to the AA group or the Both group (Ms = 0.53, 0.57, respectively). With respect to lifetime detox treatments, the SMART group had fewer treatments on average (M = 1.03) than the AA group or the Both group (Ms = 4.06, 2.92, respectively). The Neither group had fewer treatments on average (M = 0.90) than the AA group and the Both group (Ms = 4.06, 2.92, respectively). With respect to past 90-day detox treatments, the SMART group had fewer treatments on average (M = 0.21) than the Both group (M = 0.53). The Neither group had fewer treatments on average (M = 0.02) than the SMART group, the AA group, and the Both group (Ms = 0.21, 0.36, 0.53, respectively). With respect to lifetime inpatient treatment, the SMART group had fewer treatments on average (M = 0.92) compared to the AA group and the Both group (Ms = 2.85, 2.53, respectively). Similarly, the Neither group had fewer treatments on average (M = 0.77) than the AA group (M = 2.85) and the Both group (M = 2.53). With respect to past 90-day inpatient treatments, the SMART group had fewer treatments on average (M = 0.16) compared to the Both group (M = 0.43). Likewise, the Neither group had fewer treatments on average (M = 0.02) compared to the SMART group, the AA group, and the Both group (Ms = 0.16, 0.29, 0.43, respectively). With respect to the percentage of the sample using SUD or MH services in the past 90 days, the Neither group had the lowest percentage of individuals using these services (41%) compared to the SMART group, the AA group, and the Both group (76%, 74%, and 82%, respectively).

#### 3.1.3 Legal system involvement variable differences between groups at baseline

With respect to legal system involvement, the SMART group contained the smallest proportion of individuals reporting one or more lifetime arrests (47%) compared to the AA group (71%). Similarly, the Neither group had a lower proportion of individuals reporting one or more lifetime arrests (46%) compared to the AA group and the Both group (71% and 67%, respectively). The AA group also had a higher average number of lifetime arrests (M = 3.08), compared to the SMART group and the Neither group (Ms = 1.40, 1.82, respectively).

### 3.2 What are the MHO participation rates and alcohol-related outcomes across these pathway groups over 6 months after starting an AUD recovery attempt?

**Table 2** and **Figure 1** report descriptive statistics (mean, standard deviation) for alcohol and drug outcomes and meeting attendance by group (SMART, AA, Both, Neither) at baseline and the 3- and 6-month follow-ups. For all groups, alcohol-related outcomes improved in the expected salutary directions. As anticipated, with respect to meeting attendance, the AA and Both groups showed higher rates of AA attendance, while the SMART and Both groups showed higher rates of SMART attendance (although the levels of attendance were considerably lower in the SMART group). This provides support for the finding that participants’ self-selected groups (i.e., SMART, AA, Both, Neither) were consistent with their patterns of MHO meeting attendance.

**Figure 1:**
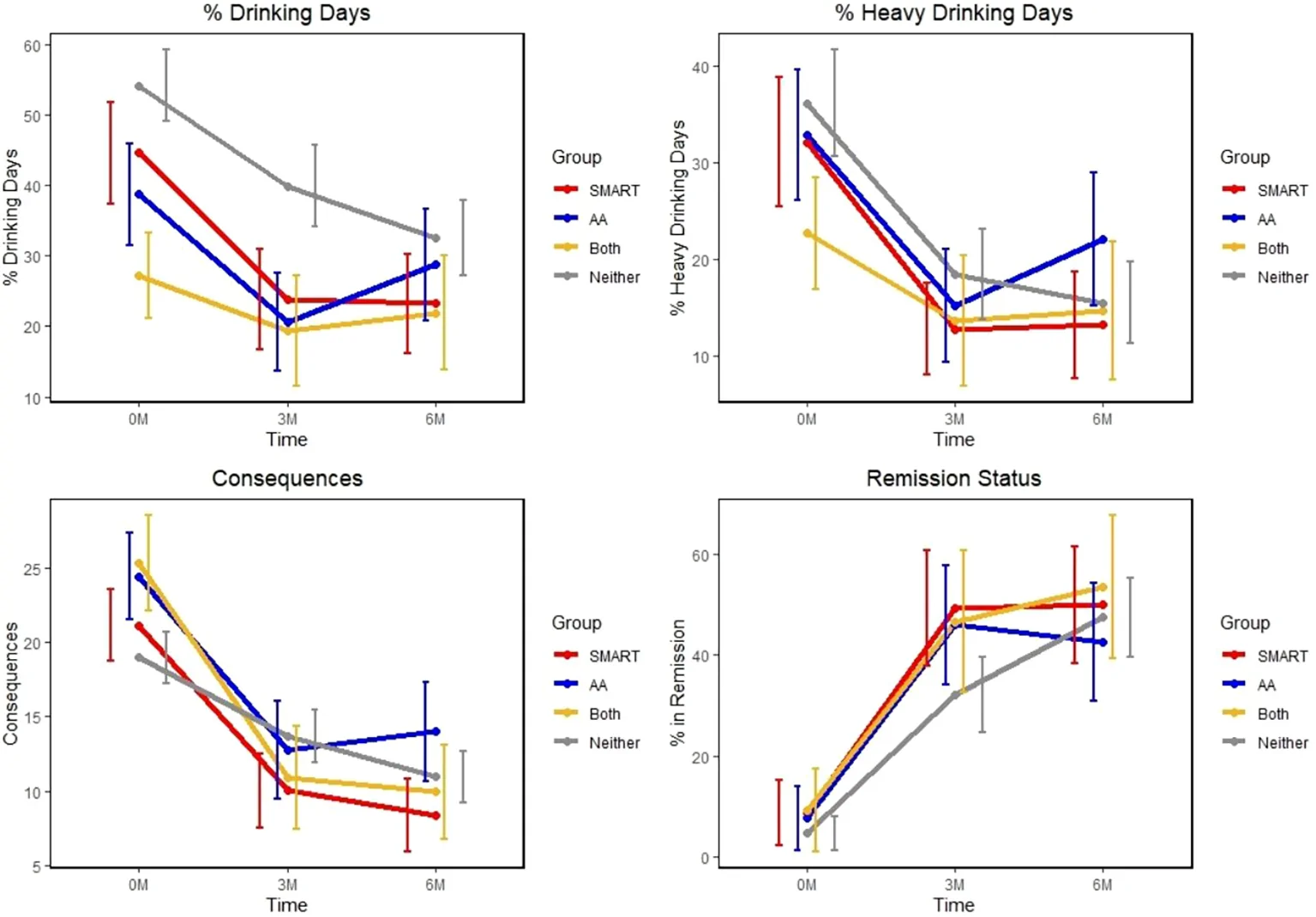
Alcohol-related outcomes across the four groups: SMART recovery, AA, both SMART and AA, and neither AA nor SMART.

### 3.3 What are the effects of pathway group choice on alcohol-related outcomes, controlling for confounding variables?

To investigate this inferential longitudinal research question, it was necessary to establish and control for predictors of study dropout and predictors of each of the four alcohol-related outcome variables to be tested in each model.

#### 3.3.1 Predictors of study dropout

First, in terms of predictors of study dropout, the proportion who dropped out (i.e., did not complete a 3- or 6-month follow-up assessment) did not differ significantly between groups (p = .42; SMART dropout rate = 4%, AA = 8%, Both = 8%, and Neither = 10%). To detect and control for variables that predicted study dropout, we screened across four sets of baseline variables: (1) *demographic variables* (i.e., age, gender, race, ethnicity, education, marital status, employment status, employment type [unemployed, part-time, full-time], and income); (*2*) *clinical severity and substance-related variables* (i.e., alcohol diagnosis/AUD symptoms, primary substance [alcohol vs. other], drinking goal, psychological distress, days bothered by mental health, days mental health interferes with responsibilities at work/school/home, alcohol craving [Penn Alcohol Craving Scale (PACS)], days drunk or high, days alcohol interferes with functioning, and prior 90-day treatment utilization variables at baseline [i.e., number of detoxes, number of inpatient treatments, and number of outpatient treatments]); (*3*) *legal system involvement variables* (i.e., lifetime criminal justice involvement and number of arrests in lifetime); and (*4*) *psychosocial variables* (i.e., religiosity and spirituality variables [daily spiritual experiences, self-report spirituality, and self-report religiosity], recovery self-efficacy, recovery capital, motivation for sobriety, coping, happiness, self-esteem, quality of life, psychological well-being, pain, and impulsivity).

Of all these potential predictors, only one variable predicted study dropout in univariate analyses (quality of life, p <.05): individuals who did not attend any study follow-up visit reported slightly lower quality of life (M = 25.7, SD = 5.61) compared to individuals who did (M = 27.0, SD = 5.60). As no additional predictors were identified, no subsequent multivariate model was needed. Thus, baseline quality of life was retained as a control variable for all subsequent models.

#### 3.3.2 Predictors of outcomes based on group difference variables at baseline

To examine predictors of outcomes based on between-group (i.e., SMART, AA, Both, Neither) differences at baseline, we screened demographic variables (i.e., gender, ethnicity, race, income, education, marital status, and location), treatment utilization variables (i.e., number of outpatient treatments, number of detox treatments, number of inpatient treatments, and % service utilization), clinical severity indicators (i.e., AUD symptoms and lifetime number of mental health diagnoses), and legal system involvement variables (i.e., lifetime number of arrests and lifetime legal system involvement). In univariate analyses assessing predictors of our four dependent variables (PDD, PHDD, % in remission, and alcohol-related consequences), we detected five significant baseline predictors of subsequent PDD (AUD symptoms, outpatient treatment, detox treatments, inpatient treatments, and % service utilization, all p <.10); two significant predictors of PHDD (ethnicity and education [high school vs. some college], all ps <.10), six significant predictors of consequences (education [high school vs. some college; high school vs. BA or higher], marital status, AUD symptoms, lifetime mental health diagnoses, and arrests in the past 90 days, all ps <.10); and four significant predictors of remission status (education [high school vs. some college], lifetime mental health diagnoses, past 90-day detox treatments, and past 90-day inpatient treatments).

In the final multivariate models, we found two significant predictors of PDD, specifically the number of detox treatments and AUD symptoms (all ps <.05), whereby individuals with more detox treatments in the past 90 days reported more drinking days at 3 and 6 months, and individuals with more baseline AUD symptoms also reported more drinking days at 3 and 6 months. With respect to PHDD, two predictors remained significant, specifically ethnicity and education, whereby non-Hispanic individuals and individuals in the lower education category (i.e., high school) had higher PHDD compared to Hispanic individuals and individuals with some college education, respectively. With respect to consequences, only one predictor remained significant—marital status, whereby married participants reported lower consequences compared to single individuals. With respect to remission status, we found two significant predictors: lifetime mental health diagnoses and past 90-day detox treatments, whereby remitted individuals reported more detox treatments and had fewer lifetime mental health diagnoses compared to those not in remission.

#### 3.3.3 Predictors of outcomes among variables not different between groups at baseline

In a similar manner, first using univariate analyses, we screened demographic variables (i.e., age, employment status, and employment type); clinical severity and substance-related variables (i.e., distress, number of days bothered by mental health, mental health interference, alcohol craving, perceived stress, psychological well-being, substance use consequences, days drunk or high, and days alcohol interfered with functioning); and psychosocial variables (i.e., abstinence self-efficacy, recovery capital, commitment to sobriety, coping, religiosity and spirituality, happiness, self-esteem, quality of life, and other variables [i.e., drinking goal, pain, and impulsivity]), placing any significant univariate predictors into a final multivariate model. In the multivariate model analyses for each dependent variable, we found three significant predictors of PDD (age, drinking goal, single-item self-efficacy, all ps <.05), whereby individuals who were older, had a more drinking-oriented drinking goal, and reported lower self-efficacy had higher PDD; one significant predictor of PHDD (single-item self-efficacy, p <.01), whereby individuals with higher self-efficacy had lower PHDD; two significant predictors of consequences (recovery capital and self-esteem, all ps <.10), whereby individuals with lower recovery capital and higher self-esteem reported greater consequences; and three significant predictors of remission status (recovery capital, self-efficacy, and craving [TPS]), whereby individuals with higher baseline recovery capital and self-efficacy and lower craving were more likely to achieve subsequent remission.

#### 3.3.4 Tests for multicollinearity and finalization of control variables for predictive models

To test for and prevent potential multicollinearity among model predictor variables, we examined bivariate relationships among predictors to see if any were highly intercorrelated before entering them in the final model. As noted above, we used a threshold of r ≥.70, as recommended (32, 33). Given concerns about skewness as discussed above, we computed both parametric (Pearson) and non-parametric (Spearman) correlation matrices. The majority of correlations between predictor variables were low (rs = 0.02–0.60); therefore, none surpassed the threshold for concern, and all were included in the models.

#### 3.3.5 Final linear mixed-effects models of group × time on outcome variables

To determine whether or how any observed relationship between our quasi-independent group variable and each of the four dependent variables changed as a result of adding the different covariates, we ran sequential incremental models, starting with a completely unadjusted model (Model 0), followed by subsequent models that added (1) predictors of dropout (Model 1); (2) Model 1 + predictors of the outcome based on baseline differences between groups (Model 2); and (3) Models 1 + 2 + any other predictors of the outcome (Model 3). Additionally, as linear mixed-effects (LME) models require specification of a reference group, we ran separate models with SMART listed as the reference group and further sets of models in which AA and Both were listed as reference groups. In order to conform to the distributional assumptions for the inferential model tests, we ran analyses using the categorized variables for PDD and PHDD.

#### 3.3.6 Percent days drinking

As shown in **Tables 3a–c**, we found significant main-effect differences in PDD between the SMART and Neither groups, the AA and Neither groups, and the Both and Neither groups whereby participants in the Neither group had higher PDD at 3- and 6-month follow-ups than the SMART, AA, and Both groups.

**Table 3a:** PDD LME group model (SMART as reference group).

	Model 0			Model 1			Model 2			Model 3		
	b	SE	t	b	SE	t	b	SE	t	b	SE	t
0m PDD	0.45***	**0.05**	**8.60**	0.44***	**0.05**	**8.07**	0.44***	**0.06**	**7.89**	0.36***	**0.05**	**6.63**
Time	-0.01	0.11	-0.12	0.00	0.10	-0.03	-0.02	0.11	-0.17	-0.02	0.11	-0.19
Group: SMART vs. AA	-0.36	0.41	-0.86	-0.25	0.40	-0.61	-0.05	0.42	-0.12	0.07	0.42	0.16
Group: SMART vs. Both	0.18	0.46	0.40	0.19	0.44	0.43	0.28	0.46	0.60	0.37	0.46	0.79
Group: SMART vs. Neither	0.88*	**0.35**	**2.49**	0.74*	0.34	2.17	0.50	0.35	1.42	0.35	0.35	1.00
Group (SMART vs AA) x Time	0.16	0.16	1.02	0.11	0.15	0.70	0.06	0.16	0.36	0.06	0.16	0.37
Group (SMART vs Both) x Time	-0.03	0.17	-0.15	-0.04	0.16	-0.23	-0.05	0.17	-0.27	-0.05	0.17	-0.28
Group (SMART vs Neither) x Time	-0.19	0.13	-1.47	-0.14	0.13	-1.11	-0.10	0.13	-0.79	-0.10	0.13	-0.75
0M Quality of Life				-0.01	0.01	-1.40	-0.02*	**0.01**	**-2.30**	-0.01	0.01	-0.63
0M Past 90 Day Detox Treatments							-0.28*	**0.12**	**-2.45**	-0.23*	**0.11**	**-2.08**
0M Consequences							-0.07**	**0.02**	**-3.50**	-0.04*	**0.02**	**-2.05**
Age										0.01*	**0.00**	**2.39**
0M Drinking Goal										-0.12**	**0.04**	**-2.96**
0M Self-Efficacy										-0.10**	**0.02**	**-4.58**

m p <.10, * p <.05, ** p <.01, *** p <.001. Bolded figures highlight statistically significant effects.

**Table 3b:** PDD LME group model (AA as reference group).

	Model 0			Model 1			Model 2			Model 3		
	b	SE	t	b	SE	t	b	SE	t	b	SE	t
0m PDD	0.45***	**0.05**	**8.60**	0.44***	**0.05**	**8.07**	0.44***	**0.06**	**7.89**	0.36***	**0.05**	**6.63**
Time	0.15	0.11	1.30	0.10	0.11	0.93	0.04	0.11	0.33	0.04	0.11	0.33
Group: AA vs. SMART	0.36	0.41	0.86	0.25	0.40	0.61	0.05	0.42	0.12	-0.07	0.42	-0.16
Group: AA vs. Both	0.54	0.46	1.17	0.44	0.45	0.98	0.33	0.47	0.70	0.30	0.47	0.64
Group: AA vs. Neither	1.23**	**0.36**	**3.39**	0.99**	**0.35**	**2.79**	0.55	0.37	1.50	0.29	0.37	0.77
Group (AA vs. SMART) X Time	-0.16	0.16	-1.02	-0.11	0.15	-0.70	-0.06	0.16	-0.36	-0.06	0.16	-0.37
Group (AA vs. Both) X Time	-0.18	0.17	-1.06	-0.14	0.17	-0.86	-0.10	0.18	-0.58	-0.11	0.18	-0.60
Group (AA vs. Neither) X Time	-0.35*	**0.14**	**-2.60**	**-0.25m**	**0.13**	**-1.87**	-0.16	0.14	-1.18	-0.16	0.14	-1.14
0M Quality of Life				-0.01	0.01	-1.40	-0.02*	**0.01**	**-2.30**	-0.01	0.01	-0.63
0M Past 90 Day Detox Treatments							-0.28*	**0.12**	**-2.45**	-0.23*	**0.11**	**-2.08**
0M Sip Consequences							-0.07**	**0.02**	**-3.50**	-0.04*	**0.02**	**-2.05**
Age										0.01*	**0.00**	**2.39**
0M Drinking Goal										-0.12**	**0.04**	**-2.96**
0M Self-Efficacy										-0.10***	**0.02**	**-4.58**

m p < .10, * p < .05 ** p < .01, *** p < .001. Bolded figures highlight statistically significant effects.

**Table 3c:** PDD LME group model (both as reference group).

	Model 0			Model 1			Model 2			Model 3		
	b	SE	t	b	SE	t	b	SE	t	b	SE	t
0M PDD	0.45***	**0.05**	**8.60**	0.44***	**0.05**	**8.07**	0.44***	**0.06**	**7.89**	0.36***	**0.05**	**6.63**
Time	-0.04	0.13	-0.29	-0.04	0.13	-0.33	-0.06	0.13	-0.48	-0.07	0.14	-0.50
Group: Both vs. SMART	-0.18	0.46	-0.40	-0.19	0.44	-0.43	-0.28	0.46	-0.60	-0.37	0.46	-0.79
Group: Both vs. AA	-0.54	0.46	-1.17	-0.44	0.45	-0.98	-0.33	0.47	-0.70	-0.30	0.47	-0.64
Group: Both vs. Neither	**0.69m**	**0.41**	**1.69**	0.55	0.40	1.39	0.22	0.42	0.53	-0.01	0.42	-0.03
Group (Both vs. SMART) X Time	0.03	0.17	0.15	0.04	0.16	0.23	0.05	0.17	0.27	0.05	0.17	0.28
Group (Both vs. AA) X Time	0.18	0.17	1.06	0.14	0.17	0.86	0.10	0.18	0.58	0.11	0.18	0.60
Group (Both vs. Neither) X Time	-0.17	0.15	-1.11	-0.10	0.15	-0.71	-0.06	0.15	-0.38	-0.05	0.16	-0.32
0M Quality of Life				-0.01	0.01	-1.40	-0.02*	**0.01**	**-2.30**	-0.01	0.01	-0.63
0M Past 90 Day Detox Treatments							-0.28*	**0.12**	**-2.45**	-0.23*	**0.11**	**-2.08**
0M Sip Consequences							-0.07**	**0.02**	**-3.50**	-0.04*	**0.02**	**-2.05**
Age										0.01*	**0.00**	**2.39**
0M Drinking Goal										-0.12**	**0.04**	**-2.96**
0M Self-Efficacy										-0.10***	**0.02**	**-4.58**

m p < .10, * p < .05 ** p < .01, *** p < .001. Bolded figures highlight statistically significant effects.

In the model where SMART was the reference group (**Table 3a**), this PDD difference was present in the unadjusted model (b = .88) and persisted after controlling for predictors of dropout (i.e., quality of life), although the magnitude of the coefficient was reduced slightly (from b = .88 to b = .74; see Model 1, **Table 3a**). In Model 2, when controlling for predictors of dropout and, additionally, baseline between-group differences that predicted PDD (i.e., past 90-day detox; baseline consequences), the initial difference between SMART and the Neither group was rendered non-significant and was further decreased in magnitude (from b = .74 to b = .50). In Model 3, controlling additionally for other baseline predictors of PDD (i.e., age, drinking goal, and self-efficacy), the between-group difference was reduced still further in magnitude (from b = .50 to b = .35).

In the model where AA was the reference group (**Table 3b**), a PDD difference was present in the unadjusted model (b = 1.23) and persisted after controlling for predictors of dropout (i.e., quality of life), although the magnitude of the coefficient was reduced (from b = 1.23 to b = .99; see Model 1, **Table 3b**). In Model 2, when controlling for predictors of dropout and, additionally, baseline between-group differences that predicted PDD (i.e., past 90-day detox; baseline consequences), the initial difference between AA and the Neither group was rendered non-significant and was further decreased in magnitude (from b = .99 to b = .55). In Model 3, controlling additionally for other baseline predictors of PDD (i.e., age, drinking goal, and self-efficacy), the between-group difference was reduced still further in magnitude (from b = .55 to b = .29).

In the model where Both was the reference group (**Table 3c**), a PDD difference was present in the unadjusted model (b = 0.69) and was rendered non-significant after including predictors of dropout (i.e., quality of life), with the magnitude of the coefficient reduced (from b = .69 to b = .55; see Model 1, Tab**le 3c**). In Model 2, when controlling for predictors of dropout and, additionally, baseline between-group differences that predicted PDD (i.e., past 90-day detox; baseline consequences), the initial difference between Both and the Neither group was further decreased in magnitude (from b = .55 to b = .22). In Model 3, controlling additionally for other baseline predictors of PDD (i.e., age, drinking goal, and self-efficacy), the between-group difference was reduced still further in magnitude (from b = .22 to b = -0.01).

#### 3.3.7 Percent heavy drinking days (PHDD)

As shown in **Tables 4a–c** and **5a, b**, we found no significant main-effect differences or interactions with time in PHDD between the SMART, AA, and Both groups and all other groups.

**Table 4a:** Percent heavy drinking days (PHDD) group model (SMART as reference Group).

	Model 0			Model 1			Model 2			Model 3		
	b	SE	t	b	SE	t	b	SE	t	b	SE	t
0M PHDD	0.28***	**0.05**	**6.04**	0.27***	**0.05**	**5.63**	0.25***	**0.05**	**5.29**	0.25***	**0.05**	**5.58**
Time	0.00	0.12	-0.02	0.00	0.12	0.00	0.00	0.12	-0.03	-0.01	0.12	-0.05
Group: SMART vs. AA	-0.11	0.45	-0.25	-0.18	0.46	-0.40	-0.19	0.46	-0.41	-0.20	0.46	-0.43
Group: SMART vs. Both	0.18	0.49	0.37	0.14	0.49	0.28	0.12	0.49	0.24	0.15	0.49	0.30
Group: SMART vs. Neither	0.41	0.38	1.08	0.35	0.38	0.91	0.39	0.39	1.01	0.17	0.39	0.43
Group (SMART vs. AA) X Time	0.13	0.17	0.73	0.13	0.18	0.73	0.14	0.18	0.78	0.15	0.18	0.84
Group (SMART vs. Both) X Time	-0.04	0.19	-0.21	-0.04	0.19	-0.19	-0.03	0.19	-0.16	-0.04	0.19	-0.22
Group (SMART vs. Neither) X Time	-0.09	0.15	-0.60	-0.08	0.15	-0.54	-0.09	0.15	-0.58	-0.08	0.15	-0.54
0M Quality of Life				**-0.02m**	**0.01**	**-1.92**	-0.02*	**0.01**	**-2.44**	0.00	0.01	-0.14
Ethnicity							**0.32m**	**0.17**	**1.93**	**0.29m**	**0.16**	**1.82**
Education: High School vs. Some College							-0.37*	**0.15**	**-2.54**	-0.39**	**0.14**	**-2.78**
Education: High School vs. College Graduate							-0.05	0.14	-0.34	-0.19	0.14	-1.35
0M Self-Efficacy										-0.11***	**0.02**	**-5.99**

m p < .10, * p < .05 ** p < .01, *** p < .001. Bolded figures highlight statistically significant effects.

**Table 4b:** Percent heavy drinking days (PHDD) group model (AA as reference Group).

	Model 0			Model 1			Model 2			Model 3		
	b	SE	t	b	SE	t	b	SE	t	b	SE	t
0M % Heavy Drinking Days	0.28***	**0.05**	**6.04**	0.27***	**0.05**	**5.63**	0.25***	**0.05**	**5.29**	0.25***	**0.05**	**5.58**
Time	0.13	0.13	0.99	0.13	0.13	0.98	0.13	0.13	1.04	0.14	0.13	1.09
Group: AA vs. SMART	0.11	0.45	0.25	0.18	0.46	0.40	0.19	0.46	0.41	0.20	0.46	0.43
Group: AA vs. Both	0.30	0.51	0.58	0.32	0.51	0.63	0.31	0.51	0.60	0.35	0.51	0.68
Group: AA vs. Neither	0.52	0.40	1.30	0.53	0.41	1.31	0.58	0.41	1.42	0.37	0.41	0.89
Group (AA vs. SMART) X Time	-0.13	0.17	-0.73	-0.13	0.18	-0.73	-0.14	0.18	-0.78	-0.15	0.18	-0.84
Group (AA vs. Both) X Time	-0.17	0.19	-0.86	-0.16	0.20	-0.84	-0.17	0.20	-0.86	-0.19	0.20	-0.97
Group (AA vs. Neither) X Time	-0.22	0.15	-1.39	-0.21	0.16	-1.33	-0.22	0.16	-1.43	-0.23	0.16	-1.46
0M Quality of Life				**-0.02m**	**0.01**	**-1.92**	-0.02*	**0.01**	**-2.44**	0.00	0.01	-0.14
Ethnicity							**0.32m**	**0.17**	**1.93**	**0.29m**	**0.16**	**1.82**
Education: High School vs. Some College							-0.37*	**0.15**	**-2.54**	-0.39**	**0.14**	**-2.78**
Education: High School vs. College Graduate							-0.05	0.14	-0.34	-0.19	0.14	-1.35
0M Self-Efficacy										-0.11***	**0.02**	**-5.99**

m p < .10, * p < .05 ** p < .01, *** p < .001. Bolded figures highlight statistically significant effects.

**Table 4c:** Percent heavy drinking days (PHDD) group model (Both as reference Group).

	Model 0			Model 1			Model 2			Model 3		
	b	SE	t	b	SE	t	b	SE	t	b	SE	t
0M % Heavy Drinking Days	0.28***	**0.05**	**6.04**	0.27***	**0.05**	**5.63**	0.25***	**0.05**	**5.29**	0.25***	**0.05**	**5.58**
Time	-0.04	0.15	-0.28	-0.04	0.15	-0.25	-0.03	0.15	-0.22	-0.05	0.15	-0.33
Group: Both vs. SMART	-0.18	0.49	-0.37	-0.14	0.49	-0.28	-0.12	0.49	-0.24	-0.15	0.49	-0.30
Group: Both vs. AA	-0.30	0.51	-0.58	-0.32	0.51	-0.63	-0.31	0.51	-0.60	-0.35	0.51	-0.68
Group: Both vs. Neither	0.23	0.44	0.52	0.21	0.44	0.48	0.27	0.45	0.61	0.02	0.45	0.05
Group (Both vs. SMART) X Time	0.04	0.19	0.21	0.04	0.19	0.19	0.03	0.19	0.16	0.04	0.19	0.22
Group (Both vs. AA) X Time	0.17	0.19	0.86	0.16	0.20	0.84	0.17	0.20	0.86	0.19	0.20	0.97
Group (Both vs. Neither) X Time	-0.05	0.17	-0.28	-0.04	0.17	-0.26	-0.06	0.17	-0.33	-0.04	0.17	-0.23
0M Quality of Life				**-0.02m**	**0.01**	**-1.92**	-0.02*	**0.01**	**-2.44**	0.00	0.01	-0.14
Ethnicity							**0.32m**	**0.17**	**1.93**	**0.29m**	**0.16**	**1.82**
Education: High School vs. Some College							-0.37*	**0.15**	**-2.54**	-0.39**	**0.14**	**-2.78**
Education: High School vs. College Graduate							-0.05	0.14	-0.34	-0.19	0.14	-1.35
0M Self-Efficacy										-0.11***	**0.02**	**-5.99**

m p < .10, * p < .05 ** p < .01, *** p < .001. Bolded figures highlight statistically significant effects.

**Table 5a:** Consequences group models (SMART as reference group). .

	Model 0			Model 1			Model 2			Model 3		
	b	SE	t	b	SE	t	b	SE	t	b	SE	t
0m Consequences	0.31***	**0.05**	**5.95**	0.25***	**0.05**	**4.59**	0.27***	**0.06**	**4.81**	0.26***	**0.05**	**4.85**
Time	-1.39	1.24	-1.13	-1.43	1.23	-1.16	-1.42	1.24	-1.15	-1.46	1.24	-1.18
Group: SMART vs. AA	-0.61	4.78	-0.13	-1.52	4.78	-0.32	-1.89	4.83	-0.39	-1.34	4.82	-0.28
Group: SMART vs. Both	-3.20	5.25	-0.61	-3.74	5.22	-0.72	-4.46	5.25	-0.85	-3.24	5.27	-0.61
Group: SMART vs. Neither	6.28	4.08	1.54	5.09	4.06	1.25	4.72	4.09	1.15	4.67	4.09	1.14
Group (SMART vs. AA) X Time	1.21	1.78	0.68	1.35	1.78	0.76	1.36	1.80	0.75	1.50	1.80	0.83
Group (SMART vs. Both) X Time	1.18	1.96	0.60	1.21	1.95	0.62	1.19	1.96	0.61	1.20	1.98	0.61
Group (SMART vs. Neither) X Time	-1.12	1.52	-0.74	-0.94	1.51	-0.62	-0.92	1.52	-0.60	-1.01	1.53	-0.66
0M Quality of Life				-0.34***	**0.11**	**-3.09**	-0.30	0.11	-2.73	-0.02	0.13	-0.18
Married: Married vs. In a Relationship							-0.23**	**2.04**	**-0.11**	0.46	1.97	0.23
Married: Married vs. Single							**2.57m**	**1.39**	**1.85**	**2.63m**	**1.35**	**1.96**
Married: Married vs. Separated / Divorced / Widowed							0.96	1.74	0.55	1.75	1.70	1.03
0M Self-Esteem										0.42	0.27	1.53
0M Recovery Capital										-0.47***	**0.09**	**-5.34**

m p < .10, ** p < .01, *** p < .001. Bolded figures highlight statistically significant effects.

**Table 5b:** Consequences group models (AA as reference group).

	Model 0			Model 1			Model 2			Model 3		
	b	SE	t	b	SE	t	b	SE	t	b	SE	t
0M Consequences	0.31***	**0.05**	**5.95**	0.25***	**0.05**	**4.59**	0.27***	**0.06**	**4.81**	0.26***	**0.05**	**4.85**
Time	-0.18	1.28	-0.14	-0.07	1.29	-0.06	-0.06	1.30	-0.05	0.04	1.31	0.03
Group: AA vs. SMART	0.61	4.78	0.13	1.52	4.78	0.32	1.89	4.83	0.39	1.34	4.82	0.28
Group: AA vs. Both	-2.59	5.33	-0.49	-2.22	5.31	-0.42	-2.56	5.35	-0.48	-1.90	5.37	-0.35
Group: AA vs. Neither	6.89	4.19	1.65	6.61	4.19	1.58	6.61	4.24	1.56	6.00	4.24	1.42
Group (AA vs. SMART) X Time	-1.21	1.78	-0.68	-1.35	1.78	-0.76	-1.36	1.80	-0.75	-1.50	1.80	-0.83
Group (AA vs. Both) X Time	-0.03	1.98	-0.01	-0.14	1.98	-0.07	-0.17	2.00	-0.08	-0.30	2.02	-0.15
Group (AA vs. Neither) X Time	-2.33	1.55	-1.50	-2.29	1.56	-1.47	-2.27	1.58	-1.44	-2.51	1.58	-1.58
0M Quality of Life				-0.34***	**0.11**	**-3.09**	-0.30**	**0.11**	**-2.73**	-0.02	0.13	-0.18
Married: Married vs. In a Relationship							-0.23	2.04	-0.11	0.46	1.97	0.23
Married: Married vs. Single							**2.57m**	**1.39**	**1.85**	**2.63m**	**1.35**	**1.96**
Married: Married vs. Separated / Divorced / Widowed							0.96	1.74	0.55	1.75	1.70	1.03
0M Self-Esteem										0.42	0.27	1.53
0M Recovery Capital										-0.47***	**0.09**	**-5.34**

m p < .10, ** p < .01, *** p < .001. Bolded figures highlight statistically significant effects.

**Table 5c:** Consequences group models (both as reference group).

	Model 0			Model 1			Model 2			Model 3		
	b	SE	t	b	SE	t	b	SE	t	b	SE	t
0m Consequences	0.31***	**0.05**	**5.95**	0.25***	**0.05**	**4.59**	0.27***	**0.06**	**4.81**	0.26***	**0.05**	**4.85**
Time	-0.21	1.51	-0.14	-0.21	1.51	-0.14	-0.23	1.51	-0.15	-0.26	1.54	-0.17
Group: Both vs. SMART	3.20	5.25	0.61	3.74	5.22	0.72	4.46	5.25	0.85	3.24	5.27	0.61
Group: Both vs. AA	2.59	5.33	0.49	2.22	5.31	0.42	2.56	5.35	0.48	1.90	5.37	0.35
Group: Both vs. Neither	9.48*	**4.72**	**2.01**	**8.83m**	**4.69**	**1.88**	**9.18m**	**4.71**	**1.95**	**7.91m**	**4.75**	**1.66**
Group (Both vs. SMART) X Time	-1.18	1.96	-0.60	-1.21	1.95	-0.62	-1.19	1.96	-0.61	-1.20	1.98	-0.61
Group (Both vs. AA) X Time	0.03	1.98	0.01	0.14	1.98	0.07	0.17	2.00	0.08	0.30	2.02	0.15
Group (Both vs. Neither) X Time	-2.30	1.75	-1.31	-2.15	1.74	-1.24	-2.11	1.75	-1.20	-2.21	1.78	-1.24
0M Quality of Life				-0.34**	**0.11**	**-3.09**	-0.30**	**0.11**	**-2.73**	-0.02	0.13	-0.18
Married: Married vs. In a Relationship							-0.23	2.04	-0.11	0.46	1.97	0.23
Married: Married vs. Single							**2.57m**	**1.39**	**1.85**	**2.63m**	**1.35**	**1.96**
Married: Married vs. Separated / Divorced / Widowed							0.96	1.74	0.55	1.75	1.70	1.03
0M Self-Esteem										0.42	0.27	1.53
0M Recovery Capital										-0.47***	**0.09**	**-5.34**

m p < .10, ** p < .01, *** p < .001. Bolded figures highlight statistically significant effects.

#### 3.3.8 Consequences

As shown in **Tables 5a–c**, we found no significant main-effect differences in consequences between the SMART and AA groups and all other groups or interactions with time.

In the model where Both was the reference group (**Table 5c**), individuals in the Both group reported significantly higher levels of consequences compared to individuals in the Neither group. This difference was present in the unadjusted model (b = 9.48) and persisted after controlling for predictors of dropout (i.e., quality of life), although the magnitude of the coefficient was reduced (from b = 9.48 to b = 8.83; see Model 1, **Table 5c**). In Model 2, when controlling for predictors of dropout and, additionally, baseline between-group differences that predicted consequences (i.e., marital status), the initial difference between the Both group and the Neither group persisted and increased in magnitude (from b = 8.83 to b = 9.18). In Model 3, controlling additionally for other baseline predictors of consequences (i.e., self-esteem and recovery capital), the between-group difference was reduced in magnitude (from b = 9.18 to b = 7.91).

#### 3.3.9 Remission status

For remission status, as shown in **Tables 6a, b**, we found significant main-effect differences between the SMART and Neither groups and between the AA and Neither groups, whereby participants in the Neither group had a lower rate of remission at 3- and 6-month follow-ups than the SMART and AA groups.

**Table 6a:** Remission group models (SMART as reference group).

	Model 0			Model 1			Model 2			Model 3		
	b	SE	z	b	SE	z	b	SE	z	b	SE	z
0M Remission Status	**1.47m**	**0.79**	**1.87**	**1.41m**	**0.82**	**1.73**	**1.50m**	**0.81**	**1.84**	**1.40m**	**0.73**	**1.92**
Time	-0.16	0.51	-0.31	-0.20	0.52	-0.39	-0.21	0.52	-0.40	-0.24	0.52	-0.46
Group: SMART vs. AA	-0.31	1.88	-0.16	-0.27	1.94	-0.14	-0.44	1.96	-0.23	-1.04	1.92	-0.55
Group: SMART vs. Both	-1.96	2.14	-0.92	-1.99	2.19	-0.91	-2.21	2.20	-1.01	-2.64	2.22	-1.19
Group: SMART vs. Neither	-4.08*	**1.67**	**-2.44**	-3.81*	**1.72**	**-2.21**	-3.60*	**1.72**	**-2.09**	**-2.90m**	**1.69**	**-1.72**
Group (SMART vs. AA) X Time	0.04	0.72	0.05	0.08	0.75	0.10	0.12	0.75	0.15	0.18	0.74	0.24
Group (SMART vs. Both) X Time	0.84	0.83	1.01	0.91	0.84	1.08	0.93	0.85	1.10	0.91	0.86	1.06
Group (SMART vs. Neither) X Time	1.35*	**0.64**	**2.12**	**1.27m**	**0.65**	**1.95**	**1.28m**	**0.66**	**1.94**	**1.21m**	**0.65**	**1.84**
0M Quality of Life				0.06	0.04	1.59	0.04	0.04	1.05	-0.11*	**0.04**	**-2.59**
0M Detox Treatments							1.12*	**0.48**	**2.34**	0.65	0.43	1.51
Lifetime Mental Health Diagnoses							-0.14	0.11	-1.32	**-0.17m**	**0.10**	**-1.78**
0M Recovery Capital										0.07*	0.03	2.20
0M Self-Efficacy										0.30**	**0.10**	**3.06**
0M Craving										0.18*	0.08	2.31

m p < .10, * p < .05 ** p < .01. Bolded figures highlight statistically significant effects.

**Table 6b:** Remission group models (AA as reference group).

	Model 0			Model 1			Model 2			Model 3		
	b	SE	z	b	SE	z	b	SE	z	b	SE	z
0M Remission Status	**1.47m**	**0.79**	**1.87**	**1.41m**	**0.82**	**1.73**	**1.50m**	**0.81**	**1.84**	**1.33m**	**0.72**	**1.84m**
Time	-0.12	0.51	-0.23	-0.12	0.53	-0.23	-0.09	0.54	-0.18	-0.11	0.53	-0.20
Group: AA vs. SMART	0.31	1.88	0.16	0.27	1.94	0.14	0.45	1.96	0.23	0.97	1.91	0.51
Group: AA vs. Both	-1.66	2.16	-0.77	-1.72	2.21	-0.78	-1.76	2.22	-0.80	-1.60	2.22	-0.72
Group: AA vs. Neither	-3.77*	**1.69**	**-2.23**	-3.54*	**1.75**	**-2.02**	**-3.16m**	**1.75**	**-1.80**	-1.97	1.71	-1.15
Group (AA vs. SMART) X Time	-0.04	0.72	-0.05	-0.08	0.75	-0.10	-0.12	0.75	-0.15	-0.15	0.74	-0.20
Group (AA vs. Both) X Time	0.80	0.83	0.96	0.84	0.85	0.98	0.82	0.85	0.96	0.73	0.86	0.85
Group (AA vs. Neither) X Time	1.31*	**0.64**	**2.05**	**1.20m**	**0.66**	**1.81**	**1.16m**	**0.66**	**1.75**	1.07	0.65	1.63
0M Quality of Life				0.06	0.04	1.59	0.04	0.04	1.05	-0.12**	**0.04**	-2.63**
0M Detox Treatments							1.12*	**0.48**	**2.34**	0.62	0.43	1.45
Lifetime Mental Health Diagnoses							-0.14	0.11	-1.32	**-0.18m**	**0.10**	**-1.84m**
0M Recovery Capital										0.07*	**0.03**	2.15*
0M Self-Efficacy										0.30**	**0.10**	3.11**
0M Craving										0.18*	**0.08**	2.29*

m p < .10, * p < .05 ** p < .01. Bolded figures highlight statistically significant effects.

**Table 6c:** Remission group models (Both as reference group).

	Model 0			Model 1			Model 2			Model 3		
	b	SE	z	b	SE	z	b	SE	z	b	SE	z
0M Remission Status	**1.47m**	**0.79**	**1.87**	**1.41m**	**0.82**	**1.73**	**1.50m**	**0.81**	**1.84**	**1.30m**	**0.72**	**1.81**
Time	0.68	0.65	1.04	0.71	0.66	1.07	0.72	0.66	1.09	0.64	0.68	0.95
Group: Both vs. SMART	1.96	2.14	0.92	1.99	2.19	0.91	2.20	2.20	1.00	2.58	2.21	1.17
Group: Both vs. AA	1.66	2.16	0.77	1.72	2.21	0.78	1.75	2.22	0.79	1.43	2.21	0.64
Group: Both vs. Neither	-2.12	1.95	-1.09	-1.82	1.99	-0.91	-1.40	2.00	-0.70	-0.29	2.01	-0.14
Group (Both vs. SMART) X Time	-0.84	0.83	-1.01	-0.91	0.84	-1.08	-0.93	0.85	-1.10	-0.91	0.86	-1.07
Group (Both vs. AA) X Time	-0.80	0.83	-0.96	-0.84	0.85	-0.98	-0.81	0.85	-0.95	-0.69	0.86	-0.80
Group (Both vs. Neither) X Time	0.51	0.75	0.69	0.36	0.76	0.48	0.35	0.76	0.46	0.29	0.77	0.37
0M Quality of Life				0.06	0.04	1.59	0.04	0.04	1.05	-0.11**	**0.04**	**-2.60**
0M Detox Treatments							1.12*	**0.48**	**2.34**	0.62	0.42	1.48
Lifetime Mental Health Diagnoses							-0.14	0.11	-1.32	-0.17	0.10	-1.81
0M Recovery Capital										0.07*	**0.03**	**2.16**
0M Self-Efficacy										0.29**	**0.10**	**3.07**
0M Craving										0.18*	**0.08**	**2.34**

m p < .10, * p < .05 ** p < .01. Bolded figures highlight statistically significant effects.

We also observed significant interactions of group × time for both the SMART and AA models, as shown in **Tables 6a, b**. In the model where SMART was the reference group (**Table 6a**), we observed a significant interaction of group (SMART vs. Neither) × time, whereby the SMART group did not display significant changes in the rate of remission from the 3-month to the 6-month follow-up, but the Neither group did have a significant increase from 3 to 6 months. This difference in the change in remission status from the 3-month to the 6-month follow-up for the Neither group remained statistically significant across all models (see **Table 6a**), even after controlling for the various confounding variables. Specifically, in the unadjusted model (b = 1.35), the interaction effect persisted after controlling for predictors of dropout (i.e., quality of life), although the magnitude was reduced (from b = 1.35 to b = 1.27; see Model 2, **Table 6a**). In Model 2, when controlling for predictors of dropout and, additionally, baseline between-group differences that predicted the rate of remission (i.e., past 90-day detox; lifetime mental health diagnoses), the effect persisted and the magnitude was largely unchanged (from b = 1.27 to b = 1.28), and in Model 3, controlling additionally for other baseline predictors of remission status (i.e., baseline recovery capital, self-efficacy, and craving), significant differences in the change in the rate of remission from the 3-month to the 6-month follow-up for the Neither group were reduced slightly further in magnitude (from b = 1.28 to b = 1.21).

In the model where AA was the reference group (**Table 6b**), we observed a similar significant interaction of group (AA vs. Neither) × time, whereby the AA group did not show a significant change in the rate of remission from 3 months to 6 months, but the Neither group did. This statistically significant change in the rate of remission for the Neither group was present in the unadjusted model (b = 1.19) and persisted after controlling for predictors of dropout (i.e., quality of life), with the magnitude of the coefficient reduced (from b = 1.19 to b = 1.08; see Model 2, **Table 6b**). In Model 2, when controlling for predictors of dropout and, additionally, baseline between-group differences that predicted subsequent remission (i.e., past 90-day detox; lifetime mental health diagnoses), statistically significant differences in the change in the rate of remission from 3 to 6 months for the Neither group persisted, with the parameter estimate remaining largely unchanged (from b = 1.08 to b = 1.07). In Model 3, controlling additionally for other baseline predictors of remission status (i.e., baseline recovery capital, self-efficacy, and craving), the change in the rate of remission from 3 to 6 months for the Neither group was rendered non-significant and reduced slightly in magnitude (from b = 1.07 to b = 0.96).

In the model where Both was the reference group (**Table 6c**), we did not observe a significant interaction of group (Both vs. Neither) × time (p >.10).

## 4 Discussion

Findings here add to the existing preliminary longitudinal research regarding the clinical and public health utility of the mutual-help organization, SMART Recovery, as a potentially useful community-based and clinical-linkage resource in helping people with AUD starting a new recovery attempt. When compared to other individuals seeking recovery support through AA, both AA and SMART, or neither AA nor SMART, those utilizing SMART show initial differences in demographic characteristics, clinical pathology, and legal histories and evince broadly similar alcohol-specific and alcohol-related improvements over 6 months following the start of a new AUD recovery attempt. Findings add support for the utility of SMART as a potentially helpful recovery support resource, perhaps especially for individuals who possess certain demographic profiles and clinical and legal histories.

As described in detail previously (28), individuals electing to engage with SMART tended to be different demographically, clinically, and legally. This group was more likely to show a pattern of greater psychosocial stability (e.g., employment, marital status) and report more recovery capital (e.g., employment, education, and higher income) along with lower levels of psychopathology characterized by less severe AUD-related and alcohol specific indicators. This lower severity/higher stability AUD profile has been found in other studies that have investigated and compared SMART attendees to other MHO group attendees (e.g., 10), suggesting that SMART Recovery may be a particularly attractive option for this type of AUD phenotype (29). We have also found in a systematic qualitative investigation that people tend to be attracted to SMART Recovery because of its science-based foundations grounded in motivational and cognitive-behavioral theories (29). It is plausible that the higher levels of education among SMART attendees may also mean that they are better able to understand and accommodate more of these science-based SMART concepts.

When we examined the MHO participation rates and alcohol-related outcomes across these pathway groups over the 6 months after starting an AUD recovery attempt, all groups showed broadly similar improvements in absolute terms across time in the expected salutary direction. A noteworthy difference was that SMART-only participants attended fewer meetings overall than did participants in the AA or Both groups. This may reflect the fact that SMART operates substantially fewer meetings in most communities than AA (although SMART meetings are accessible online almost any hour of the day). In addition, given the lower indices of clinical severity and higher indices of recovery capital and psychosocial stability generally present among SMART participants, they may perceive less of a need for more intensive meeting attendance because they are not facing as many challenges and barriers internally and externally as the AA and Both cohorts. This may potentially make the maintenance or accrual of recovery-related resources more achievable for them with a lower “dose” of meeting exposure; nevertheless, it is possible SMART participants may have attended more if more in-person meetings were available and accessible.

The latter notion may be supported by the fact that, as illustrated in **Figure 1**, despite attending fewer SMART meetings overall than participants in the AA and Both groups, the SMART attendees showed similar improvements in AUD-related outcomes across the 3-month and 6-month follow-up points. The longitudinal models indicate that, after controlling for predictors of study discontinuation, baseline group differences, and other variables that predicted the specific AUD-related outcome variables, the SMART Recovery group showed improvements at least similar to those of participants electing to attend AA or both SMART and AA. Thus, findings suggest that SMART may be a useful and helpful option for recovery support and perhaps especially for individuals with the characteristic profile noted above.

Participants in the Neither group, who reported no desire to engage with either AA or SMART, appear to represent yet another AUD phenotype. Notably, similarly to the other groups, they had begun a serious AUD recovery attempt and made largely similar gains over the 6-month follow-up period. As such, more detailed investigation is needed to understand their specific salutary change processes that do not involve commonly used mutual-help organizations. The Neither group was similar in several ways to the SMART group in terms of lower clinical pathology, fewer legal history problems, and greater recovery capital and psychosocial stability, but exhibited some different trajectory outcomes compared to SMART. Specifically, in terms of PDD, the Neither group reported more drinking days across follow-ups than the SMART, AA, or Both groups. Furthermore, the attenuation of the magnitude of the parameters after adjusting for dropout predictor variables and the other variables that differed at baseline that also predicted PDD suggests that the greater consequences and higher number of recent detoxifications in the SMART and AA groups at baseline and the Both group’s lower baseline quality of life—which are all markers of greater alcohol-related severity and impairment—may have contributed to stronger personal emphases on stopping alcohol use completely or reducing more substantially among the MHO-involved groups and, therefore, may partially help explain this between-group difference in PDD. This is in keeping with the Health Belief Model of behavior change (34), wherein the recognized severity of illness can lead to stronger desires to change behavior—in this case, alcohol use.

Notably, despite the differences in psychosocial and clinical characteristics among the four groups, all groups improved similarly and substantially in heavy drinking frequency over the 6-month follow-up period. The fact that this outcome is associated with much more acute harms and hazards (e.g., accidents, injuries, mortality) than pure frequency of drinking days and that all groups were starting a new AUD recovery attempt (i.e., trying to stop completely or otherwise reduce alcohol use to eradicate any problems) may mean that all participants were strongly motivated and able to mobilize the necessary intrinsic resources to substantially reduce more consequential heavy use.

A similar pattern was also observed in terms of substantial reductions in alcohol-related consequences, which decreased at similar rates across all groups over time. The one exception was that the Both group reported significantly higher levels of consequences than the Neither group at baseline, but these levels then dropped substantially to levels similar to those of the other groups at 3- and 6-month follow-ups. The Both group, by definition, may have been suffering more as they were seeking more MHO support resources (i.e., attending both AA and SMART) to potentially alleviate that suffering, and their greater proactive participation in both AA and SMART may have led to the observed substantial decline in consequences.

In terms of the groups achieving AUD remission status over time, there were broadly similar improvements across the three MHO-involved groups, but the Neither group did not achieve as high a remission rate as quickly. Instead, the Neither group took another 3 months to reach the level of the MHO-involved groups at the 6-month follow-up. Interestingly, the addition of the control variables across all of the sequential models did not appear to explain this lagged effect in the Neither group’s remission rate when SMART was the reference group, as the effect remained statistically significant despite their inclusion. In contrast, when AA was the reference group, as shown in **Table 6b**, Model 3, findings suggest that the Neither group’s slower increase in achieving remission might have been due in part to lower baseline self-efficacy and recovery capital and higher craving in that group that might have slowed that group’s improvement. This general pattern of findings suggests that help-seeking through whatever MHO type may provide access to the kinds of social and other recovery capital that might help those starting a new AUD recovery attempt to achieve stability and remission sooner.

### 4.1 Limitations

Any conclusions or inferences from study findings should be considered carefully in light of several important study limitations. First, the lack of random assignment to the levels of the quasi-independent variables, resulting in very different baseline demographic and clinical characteristics across the four groups, together with the fact that participants self-selected into one of these four groups, means that any questions of relative efficacy of the four different chosen pathways cannot be answered. Rather, findings reflect the fact that individuals with different AUD phenotypes who *choose* to engage with these different recovery support resources all show similar and very positive associated potential benefits. In addition, given the complexity of the analyses, this paper reported only on the associations of pathway choices over time and not on the degree of attendance and active participation and involvement in the different MHO organizations. Future research will examine and report on these aspects. Finally, this study’s scientific rigor was arguably impacted by the onset of COVID-19, which affected our ability to implement bioassays to confirm self-reports of substance use across time. We also needed to expand recruitment to San Diego, CA, to increase our number of SMART group participants, who initially were thought to be sufficiently accrued from the Boston and New England area. This introduced regional variability, including meeting availability and demographics (e.g., more Hispanic individuals in the SMART group because the Hispanic population is much higher in San Diego—where a large number of SMART participants were recruited—than in Boston). Despite these differences, however, the findings were consistent with other formative research findings in this area using similar naturalistic designs (e.g., 10).

## 5 Conclusions

Despite the substantial increases in availability, accessibility, and participation in “second-wave” mutual-help organizations like SMART Recovery, little has been formally documented with scientific rigor regarding SMART’s clinical and public health utility. Findings here suggest that, on average, SMART Recovery may attract individuals with greater psychosocial stability, more recovery capital, and lower psychopathology and legal system involvement than do 12-step mutual-help groups such as AA. Although quite different in these ways, SMART participants appear to want and need the kinds of helpful ingredients on offer in SMART Recovery and may benefit to a degree similar to participants following more traditional 12-step pathways in terms of reductions in alcohol use and related consequences and the achievement of early remission over the first 6 months following the start of a new AUD recovery attempt. As such, SMART may provide another helpful recovery support option that certain individuals with AUD may find to be a particularly good fit.

## Data Availability

The raw data supporting the conclusions of this article will be made available by the authors, without undue reservation.
