# Supplementary material for: A comparison of recovery benefits associated with participation in SMART Recovery, AA, both SMART and AA, or neither, following a new alcohol use disorder recovery attempt
Source: Front Psychiatry. 2026 Oct 7;17:1886846. doi: 10.3389/fpsyt.2026.1886846 (PMC13683584; doi:10.3389/fpsyt.2026.1886846)
Supplement: Supplementary file 1 [file Table1.docx]

Supplement 1 – Measures

**Table 1** (staff-administered measures)

| **Measure** | | **Description** |
| --- | --- | --- |
| Substance Use History | | Participants answered a series of questions about 15 substances/classes of substances (hereafter simply referred to as substances) from the Global Appraisal of Individual Needs (GAIN-I; (Dennis et al., 2002)): 1) Alcohol, 2) Marijuana, 3) Heroin, 4) Methadone, 5) Buprenorphine and its formulations (e.g., suboxone), 6) Other opioids (e.g., pharmaceutical opioids), 7) Cocaine, 8) Amphetamines (including MDMA), 9) Methamphetamine, 10) Benzodiazepines, 11) Barbiturates, 12) Hallucinogens, 13) Synthetic drugs (e.g., synthetic cannabinoid like “K2” and synthetic cathinones such as “bath salts”), 14) Inhalants, and 15) Steroids, as well as “Other” (specified by participant).  At baseline, participants reported which of these substances they used 10 or more times in their life. Then for each substance endorsed, they provided information on the following from the Form-90 (Miller & Delboca, 1994): a) Age of first use; b) If they had ever used the substance regularly (i.e., at least once per week) (yes/no) and if so, the age of first regular use; c) Whether they had used the substance in the past three months (yes/no), and if so how many days out of the past 90 they used the substance and d) If they had not used the substance in the past 3 months, the date of their last use. For follow-ups, the questionnaire assessed if participants had used these substances in any capacity in the past 3 months, if they had used the substances regularly over the past 3 months, and out of how many of the past 90 days they used the substance. Participants then chose their primary substance (“drug of choice”) and secondary substance from the substances they had used (lifetime use for baseline assessment, past 3 month use for follow-ups). Finally, participants were asked for how many of the past 90 days their use of alcohol/drugs interfered with their functioning, and how many out of the past 90 days they got drunk at all or high for most of the day. |
| Alcohol Use Disorder DART | | Participants were asked this validated semi-structured interview to capture AUD status (AUD severity and withdrawal symptoms). Participants were first asked if they had consumed alcohol in the past 3 months. If the participant answered ‘yes’, the DART would pertain to the past 3 months. If the participant answered ‘no’, they would be asked if they consumed alcohol during the past 12 months, and the DART would pertain to the past 12 months. If the participant answered ‘no’ to both the questions, they would only be asked if they experienced strong urges or cravings to drink. |
| Substance Use Disorder DART | | Participants were asked about the recreational drugs/medications they used in the past 12 months. They then ranked the substances in the order that they caused problems for them. The DART was administered for the top 3 substances that caused the most problems for participants. |
| NIH PhenX Toolkit | | Participants reported on lifetime use of cigarettes, e-cigarettes, or another tobacco/nicotine product (specified by participant). For those who smoked cigarettes, they reported on the following: 1) Age of first regular use; 2) Of how many of the past 30 days they smoked cigarettes 3) Average number of cigarettes smoked per day in the past 30 days 4) Whether or not they had ever made a serious attempt to quit smoking. This was asked pertaining to ‘lifetime’ for the baseline visit and for the past 90 days in the follow-up visits. If participants had made a serious quit attempt either in their life (baseline) or in the past 90 days (follow-up), they reported how old they were when they most recently quit smoking, the number of quit attempts in the past 90 days, the longest length of time they had quit smoking for, as well as on psychosocial smoking cessation resources. If not currently still smoking, participants reported the age when they stopped smoking, as well as psychosocial smoking cessation resources used in their most recent quit attempt. Finally, regarding smoking cessation, all participants answered a single item with four multiple choice options to gauge attitudes toward inclusion of smoking cessation in AOD treatment (e.g., “Services that help people stop smoking…should be automatically included in addiction treatment.”). (National Cancer Institute, 2009; National Institutes of Health & U.S. Food and Drug Administration, 2013; Prorok et al., 2000). |
| Treatment for Injuries or Physical Health Problems | | Participants reported treatment in an emergency room and admissions to a hospital for at least one night for health problems. At baseline, participants reported for both lifetime and past 3 months. At follow-up visits, participants were asked to report only on treatments in the past 3 months (Dennis et al., 2002; Miller & Delboca, 1994). |
| Treatment for Alcohol and Drug Use Problems | | Participants reported treatment in an emergency room and admissions to a hospital for at least one night for alcohol or drug use problems. At baseline, participants reported for both lifetime and past 3 months. At follow-up visits, participants were asked to report only on treatments in the past 3 months (Dennis et al., 2002; Miller & Delboca, 1994). |
| Anti-craving and Anti-relapse Medications (Alcohol and Opioids) | | At baseline, participants reported whether they had ever been prescribed a medication to prevent them from drinking alcohol or using opioids. At follow-up visits, participants were asked if they had been newly prescribed any medication to prevent them from drinking alcohol or using opioids in the past 3 months. If participants responded yes to either item, participants reported lifetime (baseline), past 3 months (follow-up) and current (baseline and follow-up) use of specific medications from the Form-90, including both generic and brand names (Miller & Delboca, 1994). Participants were also asked to rate what proportion of the time they used each medication as medically indicated. |
| Mental and Emotional Health: Diagnoses, Hospitalizations, Treatment History | | Participants reported whether they had ever been told that they had a mental health condition by a doctor, nurse, or counselor, including agoraphobia, anorexia nervosa, bipolar disorder, bulimia nervosa, delusional disorder, dysthymic disorder, generalized anxiety disorder, major depressive disorder, obsessive-compulsive disorder, panic disorder, personality disorder, post-traumatic stress disorder, schizoaffective disorder, schizophrenia, social anxiety disorder, specific phobia, substance use disorder, and other. For each diagnosis endorsed, participants indicated whether this had been a problem for them in the past 12 months.  Participants reported treatment in an emergency room and admissions to a hospital for at least one night for mental, emotional, behavioral, or psychological problems. Participants reported for both lifetime and past 3 months. Participants also reported the number of times they had seen a mental health doctor in an office or outpatient clinic (including telehealth) in the past 3 months, on how many of the past 90 days they had been bothered by mental, emotional, behavioral, or psychological problems, and on how many of the past 90 days these problems had kept them from meeting their responsibilities or made them feel like they could not go on (Dennis et al., 2002; Miller & Delboca, 1994). |
| Mental and Emotional Health: Psychiatric Medication Use | | Participants were asked if they had ever been prescribed medication by a physician or medical practitioner to help them with a mental health condition (lifetime use). If they said yes, they were asked which medication they had ever been prescribed with the options antidepressants, anti-anxiety medication, anti-psychotics, mood stabilizers, stimulants, painkillers, medications for sleep and other (to be specified). Participants were then asked if they were still taking the medicines they indicated. If participants were still taking the medicines, they were asked what proportion of the time they take the prescribed medication as medically indicated. At follow-up visits, participants were asked if they were still using any medication that they had been using at the prior study visit, whether they had been newly prescribed any medication to help with a mental health condition, and if yes, what type of medication. For each medication endorsed, participants reported what proportion of the time they used the medication as medically indicated (Dennis et al., 2002; Miller & Delboca, 1994). |
| Social Support Questionnaire (SSQ) | | Participants were asked to list the initials of up to 5 family members, up to 5 friends, and up to 3 other important people in their life who they felt close to. For each person, they listed their initials, relationship, alcohol use pattern, drug use pattern, days per month they had contact with this person over the past 3 months (including contact via phone/text), how much they value their relationship on a scale of 1 (*not at all*) to 10 (*a great deal*) and how helpful they are in their recovery efforts on a scale of 1 (*not at all helpful*) to 10 (*a great deal*) (Zywiak et al., 2009). |
| 12-step/MHO Attendance History | | At baseline, participants were asked about lifetime attendance to help with their AOD problem at 12 different MHOs, with an “other” option specified by participant (Kelly et al., 2011): 1) Alcoholics Anonymous (AA); 2) Narcotics Anonymous (NA); 3) Marijuana Anonymous (MA); 4) Cocaine Anonymous (CA); 5) Crystal Methamphetamine Anonymous (CMA); 6) SMART Recovery; 7) LifeRing Secular Recovery; 8) Moderation Management; 9) Celebrate Recovery; 10) Women for Sobriety; and 11) Secular Organization for Sobriety (S.O.S.).; 12) Dual Diagnosis Anonymous (DDA); and 13) Other. Other options were examined for possible inclusion in existing categories, and recategorized as appropriate. At follow-ups, participants reported attendance for the past 3-months. For each MHO attended, participants reported a) Whether they attended regularly (at least once per week), b) Number of meetings in the past 3 months, and c) Whether they had ever attended a meeting online. |
| Online Resources and Social Network Sites | | For each MHO that a participant had attended online (as noted in “12-step/MHO Attendance History”), participants reported how many meetings they had attended online in the past 3 months, how they accessed these meetings (video, audio only, telephone, etc.), and how helpful they felt the online meetings were on a scale of 1 to 10. Participants also reported whether they used any online or mobile technologies to support their AOD problem resolution or recovery in their lifetime (baseline) and in the past 3 months. Potential online and mobile technologies included recovery-focused social network sites (e.g., InTheRooms.com), general social network sites (e.g., Facebook), and mobile smartphone applications. For each online or mobile technology endorsed, participants indicated how many of the past 90 days they had used the technology for recovery and how helpful they found it on a scale of 1 to 10. Participants were also given the opportunity to provide any other information or comments on their use of online or mobile recovery resources. |
| SMART Involvement | | Questions about SMART involvement were asked for participants who had attended SMART Recovery. Participants were asked how long they have been attending SMART Recovery, how they heard about SMART Recovery, whether they consider themselves to be a current member of SMART Recovery (*yes/no*), how many times they have attended SMART recovery in their lifetime (*numerical value*) and if another member of SMART Recovery served as a personal mentor or guide to them in the past 3 months (*yes/no*). The participants were asked questions about their participation in SMART meetings in the past 3 months to gauge the frequency of the use of SMART meetings, tools, website, and web-application ‘Overcoming Addictions.’ Participants were also asked four questions about their level of engagement in SMART meetings. |
| Recovery/Abstinence time | | Participants were asked to report in years and months how long they had been either 1) Sober (not using any alcohol/drugs) or 2) Drinking/using drugs without problems. |
| Multidimensional Mutual-Help Activity Scale (MM-HAS) | | Questions from the MM-HAS were asked for participants who had attended meetings of any of the following MHOs: Alcoholics Anonymous, Narcotics Anonymous, Marijuana Anonymous, Cocaine Anonymous, Crystal Methamphetamine Anonymous, and Dual Diagnosis Anonymous. For each organization, participants were asked if they currently considered themselves to be a part of the MHO, their activities as part of the MHO in the past 3 months (sponsor, contact with sponsor outside a meeting, contact with other members outside a meeting, read 12 step literature outside of a meeting, shared or talked during meetings, helped with setting up/running a meeting), and the number of steps out of the 12 step program that they completed while participating in the MHO in the past 3 months. Participants were then asked to rate the helpfulness, enjoyability, and safety of the MHO from a scale of 1 (*not at all helpful, do not enjoy at all,* or *not at all safe*) to 10 (*extremely helpful, enjoy a great deal,* or *completely safe*) (Kelly et al., 2013; Kelly et al., 2011). |
| Recovery support services and formal treatment programs (RSSTX) | | The questionnaire assessed history of participation in nine psychosocial treatment and recovery support services: 1) Sober living environment; 2) Recovery high school; 3) College recovery program/community 4) Recovery community center (RCC); 5) Faith-based recovery services (e.g., a recovery group provided by a church, synagogue, mosque, etc.); 6) State or local recovery community organization (RCO); 7) Outpatient addiction treatment; 8) Alcohol/drug detoxification services; 9) Inpatient or residential treatment. If they responded yes to any treatment service (7, 8 or 9), they reported the number of times they used the service (i.e., number of treatment episodes) in their lifetime (baseline) and the past 3 months (baseline and follow-up). (Dennis et al., 2002; Miller & Delboca, 1994). |
| Year End Summary (YES) | | At baseline, 12-month follow-up, and 24-month follow up, participants were asked if they felt like they were better off now than they were 12 months ago in terms of their alcohol and drug problems (worse off, same, better off). Participants were then asked to elaborate on the reason for that answer. Participants were asked if their substance use had changed or stayed the same in the past 12 months (changed for the better, changed for the worse, stayed the same). If participants reported ‘stayed the same’, they were asked what factors they thought were most responsible for their substance use staying the same. |
| Timeline Follow Back (TLFB) | | Participants provided specific dates for alcohol use, substance use, MHO attendance, inpatient and outpatient addiction treatment, inpatient and outpatient mental health treatment, and incarceration for the past 3 months (baseline, 3-, 6-, 9-, and 12-month follow-ups) or 6 months (18- and 24-month follow-ups) (Miller & Delboca, 1994). For alcohol use, participants were asked to report the number of standard drinks consumed on each drinking day. For MHO attendance and outpatient treatment for addiction and mental health, participants were asked to report whether services were in-person or online. During in-person study visits, a printed calendar was used to facilitate the TLFB. For remote visits, study staff prompted participants with potentially memorable dates within the timeframe (e.g., holidays). |
| Go/No-Go Cognitive Measure* | The Inquisit Go/No-Go Cognitive Measure is a computerized task used to assess impulsivity. The Inquisit script implemented the Go/No-Go Task as described in Fillmore et al. (2006). Participants were asked to press the spacebar when they see a green rectangle (*go*) but refrain from pressing the spacebar when they see a blue rectangle (*no-go*). The blue and green rectangles could be vertical or horizontal. The vertical rectangle had a high probability (4:1) of being green (*go*) and the horizontal rectangle had a high probability (4:1) of being blue (*no-go*). Participants were given information about the orientation of the rectangle shortly before the color of the rectangle was revealed. Participant response times and error rates were recorded. For remote visits, a web-based version of the Go/No-Go Task was tested, but due to the effect of internet speed on results, this measure was not included. | |
| Breathalyzer* | | Breathalyzer tests were used to establish a baseline level of substance use for participants at the first assessment and to ensure that data was not collected from participants who were impaired due to alcohol use. Breathalyzer tests were performed at baseline and all follow-up time points prior to starting each assessment. If a participant’s BAC was above .02, study staff did not conduct the study visit; instead, study staff either waited with the participant until their BAC dropped to .02 or lower or attempted to re-schedule the participant’s visit. If a participant’s BAC was above the legal limit (.08), and the participant had driven to their assessment, study staff asked to hold the participant’s car keys while waiting for their BAC to drop below the legal limit. If the participant insisted on holding their car keys and/or driving, or if the participant did not stay with study staff until their BAC dropped below .08, study staff called security as a safety precaution. Study staff also offered to arrange and pay for a cab to transport the participant home. If a participant had driven to the appointment and decided to take a cab home, the participant could return to pick up their car keys during business hours when their BAC was below .08. For remote visits, study staff were unable to perform breathalyzer tests and instead asked participants to verify that they had not used alcohol or other drugs prior to the assessment via self-report. |
| Urine Drug Screen* | | Urine drug screens were used to establish baseline substance use at baseline and to verify self-reported estimates of alcohol and other drug use. For remote visits, study staff were unable to perform urine drug screens. |
| Saliva Test | | Self-administered saliva tests were implemented in March 2021 as an alternative method of biochemical verification during remote visits. Participants were contacted prior to their assessment to confirm willingness to participate in the saliva test, what address the test would be mailed to, and whether the visit would be conducted over Zoom or on the phone. During the assessment, participants were able to self-administer the saliva test with study staff guidance. If participants completed the assessment over Zoom, participants showed the test results to the research coordinator who screenshotted the test and uploaded it to REDCap. If the participant completed the assessment over the phone, they uploaded images of the test to REDCap or, if unable to upload images, self-reported the results. Due to inconsistencies in saliva test results (no results, partial results, and false negatives), saliva test use was discontinued in May 2021. |

* = not administered during remote visits

**Table 2** (self-administered measures)

| **Measure** | **Description** |
| --- | --- |
| Demographics Background | Participants reported the following: gender, race, ethnicity (whether participants were Hispanic/Latino), where they were living for the majority of the past 3 months (with family or other relatives, with group of friend(s) or non-family members, alone in own dwelling, homeless, hospital rehabilitation facility or nursing home, jail, prison or other correctional facility, other), current marital status (single, married, living with someone as if married, in a relationship, engaged to be married, legally separated, divorced, widowed), sexual orientation, left or right handed, highest level of schooling completed, highest level of schooling completed by either parent, whether they held a job in the past 3 months, (if yes) nature of employment (odd jobs, part time, full time), (if no) reason for unemployment, major source of financial support, total annual household income, type of health insurance, and financial well-being of their family. Numerical values were collected for the following in the past 3 months: unplanned absences from work/school, times disciplined on the job/at school, times your job/school has been in jeopardy, times you were suspended or fired from work/school (Dennis et al., 2002; Miller & Delboca, 1994). |
| Criminal Justice Involvement | The questionnaire used adapted items about criminal justice involvement from the Form-90 (Miller & Delboca, 1994). Participants reported on their current legal status (none, on probation only, on parole only, on probation and parole, awaiting charge, trial or sentence, outstanding warrant, case pending, other). At baseline, participants reported whether they had ever been arrested (yes/no). If yes, they reported how many times overall, how many times for DUI/DWI in their lifetime, and how many times for other reasons in their lifetime. At follow-up visits, participants reported whether they had been arrested in the past 3 months (yes/no). If yes, they reported how many times overall, how many times for DUI/DWI in the past 3 months, and how many times for other reasons in the past 3 months. At baseline, participants reported whether they had ever stayed in jail or prison overnight or longer (yes/no). If yes, they reported how many times in their lifetime and how many times in the past 3 months. At follow-up visits, participants only reported the number of times in the past 3 months. |
| Religious Background and Behaviors (RBBS) | At baseline, the questionnaire assessed if the participant considered themselves to be part of a religious group (No/none, Baptist, Buddhist, Catholic, Evangelical, Hindu, Jewish, Lutheran, Methodist, Mormon, Muslim, Presbyterian, Other Protestant, Shinto, Native American Church, Traditional Native American, Christian, Some other group). For all timepoints participants were asked which of the following describes them at this time: atheist, agnostic, unsure, spiritual, religious. Participants were asked how often they participated in religious activities in the past 3 months on a 7-point Likert scale (*never, rarely, once a month, twice a month*, *once a week, twice a week, almost daily, more than once a day*) and, at baseline, how often they participated in certain religious activities in their lifetime on a 3 point-Likert scale (*never, yes, in the past but not now, yes, and I still do*) (Connors et al., 1996). |
| Religious and Spiritual Intensity | The questionnaire included four items assessing participants’ religiosity and spirituality. Participants reported the extent to which they considered themselves religious/spiritual on a Likert scale from *not religious/spiritual at all* (1) to *very religious/spiritual* (4). Participants reported the extent to which their religious/spiritual practices and beliefs help them with resolving an alcohol/drug problem on a scale from *do not help at all* (1) to *make all the difference* (5) (Idler et al., 2003). |
| Twelve Promises Scale (TPS) | This questionnaire assessed participants’ current psychosocial state and attitudes towards drinking and using drugs. Participants rated how true each item was for them at the current time on a scale of *never true* (1) to *true most of the time* (5) (Kelly & Greene, 2013). |
| Perceived Stress Scale (PSS-4) | This questionnaire assessed participants’ level of stress over the last month. Participants rated each item on a scale from *never* (1) to *very often* (5) (Warttig et al., 2013). |
| Kessler 6 (K6) | This six-item scale assessed psychiatric symptoms (also referred to as psychological distress). On a scale from *all of the time* (1) to *none of the time* (5), participants are asked how often they felt: nervous, hopeless, restless or fidgety, so depressed that nothing could cheer you up, that everything was an effort, and worthless (Kessler et al., 2003). |
| Coping Strategies Scale (CSS) | Participants were asked to select how often they used a variety of coping strategies or thoughts in the past 3 months to help them not use alcohol or drugs. Participants rated each item on a scale from *never* (1) to *frequently* (4) (Litt et al., 2003; Prochaska et al., 1988). |
| Alcohol and Drug Abstinence Self Efficacy (A-DSES-20) | Participants were asked about their feelings of confidence to not drink or use drugs in various situations in the past week. Participants rated their level of confidence for each scenario on a scale of *not at all confident* (1) to *extremely* (5) (Diclemente et al., 1994). |
| Penn Alcohol and Drug Craving (PADCS-5) | This questionnaire assessed the frequency and strength of cravings to use alcohol and other drugs during the past week. Participants reported how often they thought about drinking/using drugs, how strong the craving was at its most severe, how much time they have spent thinking about drinking/using drugs, how difficult it would have been to resist drinking/using drugs, and then rated their overall alcohol/drug craving with options ranging from *never thought about drinking/using drugs and never had the urge to drink/use drugs* to *thought about drinking/using drugs nearly all of the time and had the urge to drink/use drugs nearly all of the time* (Flannery et al., 1999). |
| Commitment to Sobriety Scale (CSS-5) | In this questionnaire, participants were asked 5 questions about their commitment to not using alcohol/drugs. Participants rated the extent to which they agreed with these statements on a scale from *strongly disagree* (1) to *strongly agree* (6) (Kelly & Greene, 2014). |
| Drinking Goal | In this questionnaire, participants chose one goal that was the most true to them currently from the 5 options: 1) Total abstinence; never use again; 2) Total abstinence; but realize a slip is possible; 3) Occasional use when urges strongly felt; 4) Temporary abstinence; or 5) Controlled use. |
| Short Inventory of Problems (SIP-2R) | This questionnaire assessed how often participants had experienced various problems during the past 3 months because of their drinking/drug. Participants indicated how often they had experienced each problem on a scale of *never* to *daily or almost daily*. Participants were also asked to indicate whether they had had an accident while drinking or intoxicated in the past 3 months (Miller et al., 1995). |
| Questions about Recovery | This questionnaire assessed recovery identity, definition, and what participants believe are the factors helping them resolve their problem with alcohol/drugs at baseline, 12-month follow-up, and 24-month follow-up. Participants selected a statement that best applied to them from whether they consider themselves to be *in recovery*, *seeking recovery* or *not in or seeking recovery*. If participants chose that they were ‘in recovery’, they were asked to provide the date they use to mark the beginning of their recovery. Participants were asked to provide their definition of recovery in one sentence (free response) and to select one of three statements that best fit their definition of recovery: 1) Abstinence from all drugs/alcohol; 2) Abstinence from only those drugs/alcohol with which they had a problem; or 3) Non-problematic/moderate use of drugs/alcohol, including those with which they had a problem. Participants were then asked to list the top 3 things that have helped or are helping them to resolve their problem with alcohol/drugs. |
| Brief Assessment of Recovery Capital (BARC-10) | The BARC-10 (Vilsaint et al., 2017) is a 10-item, abridged version of the Addiction Recovery Capital Scale (Groshkova et al., 2013). The BARC-10 measures personal (e.g., “I take full responsibility for my actions”), social (e.g., “I get lots of support from friends”), physical (e.g., “I have enough energy to complete the tasks I set for myself”), and environmental resources (e.g., “My living space has helped to drive my recovery journey”) used to initiate and sustain recovery. Participants rated their agreement with each statement on a scale from *strongly disagree* (1) to *strongly agree* (6). |
| Behavioral Addictions | The questionnaire used items adapted from Laudet et al. (2015) to assess whether individuals ever had a problem with one or more other behaviors apart from AOD use in their lifetime (baseline) and currently, including 1) Eating disorder; 2) Sex/love addiction; 3) Gambling; 4) Video gaming addiction; 5) Compulsive shopping; 6) Internet addiction (for issues not assessed by other choices); 7) Compulsive exercise; 8) Internet pornography addiction; 9) Self-harm/injury and 10) Other (specified). "Other" options were examined for possible inclusion in existing categories, and recategorized as appropriate. Participants were asked if any reported behavioral addictions had been a problem for them in the past 3 month and if so, how many days out of 90. If reporting more than one, participants indicated which behavior had been the most problematic. |
| Medical Marijuana Use | Participants were asked if they had ever been recommended to use marijuana for medical reasons. If yes was indicated, participants were asked how many days out of the past 90 marijuana was used for medical reasons and to list up to three medical reasons for using marijuana. |
| Medication Attitudes | This questionnaire assessed participant attitudes toward medication for an alcohol problem, opioid problem, any kind of alcohol/drug problem, and emotional problem. Participants rated their agreement with the use of medication for these problems on a scale of *strongly disagree* (1) to *strongly agree* (6). |
| Impulsive behavior (SUPPS-S) | This questionnaire assessed impulsivity. Participants rated their agreement with 20 items describing situations or feelings related to impulsivity on a scale of *agree strongly* (1) to *disagree strongly* (4) (Coskunpinar et al., 2013). |
| Quality of Life (Q-LES-Q) | This measure of quality of life was used to assess satisfaction related to physical health, mood, relationships, activities, and economic status. Participants rated their satisfaction with each item during the past week on a scale of *very poor* (1) to *very good* (5) (Endicott et al., 1993). |
| Quality of Life (EQ5D3L) | This measure of quality of life was used to assess physical and mental health states. Participants rated their current mobility, self-care, usual activities, pain/discomfort, and anxiety/depression. Participants also rated their current overall physical and mental health states using a visual analogue scale with options between 0 (*worst*) and 100 (*best*) (Devlin & Brooks, 2017). |
| Quality of Life (EUROHIS-QOL) | This measure of quality of life is a widely used eight-item measure of quality of life, adapted from the World Health Organization Quality of Life – Brief Version (WHOQOL-BREF) (Schmidt et al., 2006). Participants rated each item on a 5-point Likert scale from 1 (*very poor, very dissatisfied,* or *not at all*) to 5 (*very good, very satisfied,* or *completely*) (da Rocha et al., 2012). |
| Pittsburgh Sleep Quality Index (PSQI) | This questionnaire assessed quality of sleep. Participants reported how many hours of sleep they got on average per night over the past month. Participants then rated their quality of sleep on a scale from *very good* (1) to *very bad* (4) (Buysse et al., 1989). |
| Pain Visual Analogue Scale (VAS) | This measure assessed physical pain. Participants rated the current severity of their pain using a visual analogue scale with options between 0 (*no pain*) to 100 (*very severe pain*) (Wewers & Lowe, 1990). |
| International Physical Activity Questionnaire (IPAQ) | This questionnaire asks participants about their level of physical activity over the past seven days. Participants indicate how many days in the past 7 days they have done: vigorous physical activity, moderate physical activity, and walking. Participants then indicate how much time per day they usually spent on each activity in hours and minutes. Participants are also asked how many hours they usually spent sitting on weekdays over the past 7 days (Hagstromer et al., 2006). |
| Meals | Participants reported how many meals on average they have eaten per day during the past 3 months. |
| Self-esteem, Happiness, and Satisfaction with Life | Three single-item measures were used to assess self-esteem (Robins et al., 2001), happiness, and satisfaction with life (Diener et al., 1985). For self-esteem, participants indicated their agreement with the statement “I have high self-esteem” on a scale from 1 (*not very true of* me) to 10 *(very true of* me). For happiness, participants rated how happy they were with their life in general on a scale of 1 (*completely unhappy*) to 10 (*completely happy*). For satisfaction with life, participants indicated their agreement with the statement “I am satisfied with my life” on a scale of *strongly disagree* (1) to *strongly agree* (7). |
| Abstinence Self-Efficacy Single Item | Participants rated how confident they were that they could remain abstinent or drink/use drugs without problem in the next 3 months on a scale from *not at all confident* (1) to *very confident* (10). |
| Daily Spiritual Experiences Scale (DSES) | This questionnaire assesses spiritual and/or religious experiences. The questionnaire includes items with the word “God” used but includes instructions for participants that if “God” is not a comfortable word that they should substitute it for one that calls to mind the divine and holy for them. Participants read 15 items describing spiritual and/or religious experiences that a person may have and rate how often they have this experience from *many times a day* (1) to *never or almost never* (6). The last item asks participants how close they feel to God from *not close* (1) to *as close as possible* (4) (Underwood & Teresi, 2002) |

**References**

Buysse, D. J., Reynolds, C. F., 3rd, Monk, T. H., Berman, S. R., & Kupfer, D. J. (1989). The Pittsburgh Sleep Quality Index: a new instrument for psychiatric practice and research. *Psychiatry Res*, *28*(2), 193-213. <https://doi.org/10.1016/0165-1781(89)90047-4>

Connors, G. J., Tonigan, J. S., & Miller, W. R. (1996). A Measure of Religious Background and Behavior for Use in Behavior Change Research. *Psychology of Addictive Behaviors*, *10*(2), 90-96.

Coskunpinar, A., Dir, A. L., & Cyders, M. A. (2013). Multidimensionality in impulsivity and alcohol use: a meta-analysis using the UPPS model of impulsivity. *Alcohol Clin Exp Res*, *37*(9), 1441-1450. <https://doi.org/10.1111/acer.12131>

da Rocha, N. S., Power, M. J., Bushnell, D. M., & Fleck, M. P. (2012). The EUROHIS-QOL 8-item index: comparative psychometric properties to its parent WHOQOL-BREF. *Value Health*, *15*(3), 449-457. <https://doi.org/10.1016/j.jval.2011.11.035>

Dennis, M. L., Titus, J., White, M., Unsicker, J., & Hodkgins, D. (2002). Global Appraisal of Individual Needs (GAIN): Administration Guide for the GAIN and Related Measures. In. Bloomington, IL: Chestnut Health Systems.

Devlin, N. J., & Brooks, R. (2017). EQ-5D and the EuroQol Group: Past, Present and Future [Review]. *Applied Health Economics and Health Policy*, *15*(2), 127-137. <https://doi.org/10.1007/s40258-017-0310-5>

Diclemente, C. C., Carbonari, J. P., Montgomery, R. P. G., & Hughes, S. O. (1994). The Alcohol Abstinence Self-Efficacy Scale [Article]. *Journal of Studies on Alcohol*, *55*(2), 141-148. <https://doi.org/10.15288/jsa.1994.55.141>

Diener, E., Emmons, R. A., Larsen, R. J., & Griffin, S. (1985). The Satisfaction With Life Scale [Article]. *Journal of Personality Assessment*, *49*(1), 71-75. <https://doi.org/10.1207/s15327752jpa4901_13>

Endicott, J., Nee, J., Harrison, W., & Blumenthal, R. (1993). Quality of Life Enjoyment and Satisfaction Questionnaire - A New Measure [Article]. *Psychopharmacology Bulletin*, *29*(2), 321-326. <Go to ISI>://WOS:A1993MC72000028

Fillmore, M. T., Rush, C. R., & Hays, L. (2006). Acute effects of cocaine in two models of inhibitory control: implications of non-linear dose effects [Article]. *Addiction*, *101*(9), 1323-1332. <https://doi.org/10.1111/j.1360-0443.2006.01522.x>

Flannery, B. A., Volpicelli, J. R., & Pettinati, H. M. (1999). Psychometric properties of the Penn Alcohol Craving Scale [Article]. *Alcoholism-Clinical and Experimental Research*, *23*(8), 1289-1295. <https://doi.org/10.1111/j.1530-0277.1999.tb04349.x>

Groshkova, T., Best, D., & White, W. (2013). The Assessment of Recovery Capital: Properties and psychometrics of a measure of addiction recovery strengths [Article]. *Drug and Alcohol Review*, *32*(2), 187-194. <https://doi.org/10.1111/j.1465-3362.2012.00489.x>

Hagstromer, M., Oja, P., & Sjostrom, M. (2006). The International Physical Activity Questionnaire (IPAQ): a study of concurrent and construct validity [Article]. *Public Health Nutrition*, *9*(6), 755-762. <https://doi.org/10.1079/phn2005898>

Idler, E. L., Musick, M. A., Ellison, C. G., George, L. K., Krause, N., Ory, M. G., Pargament, K. I., Powell, L. H., Underwood, L. G., & Williams, D. R. (2003). Measuring multiple dimensions of religion and spirituality or health research - Conceptual background and findings from the 1998 General Social Survey [Article; Proceedings Paper]. *Research on Aging*, *25*(4), 327-365. <https://doi.org/10.1177/0164027503025004001>

Kelly, J. F., & Greene, M. C. (2013). The Twelve Promises of Alcoholics Anonymous: Psychometric measure validation and mediational testing as a 12-step specific mechanism of behavior change [Article]. *Drug and Alcohol Dependence*, *133*(2), 633-640. <https://doi.org/10.1016/j.drugalcdep.2013.08.006>

Kelly, J. F., & Greene, M. C. (2014). Beyond motivation: Initial validation of the commitment to sobriety scale [Article]. *Journal of Substance Abuse Treatment*, *46*(2), 257-263. <https://doi.org/10.1016/j.jsat.2013.06.010>

Kelly, J. F., Stout, R. L., & Slaymaker, V. (2013). Emerging adults' treatment outcomes in relation to 12-step mutual-help attendance and active involvement [Article]. *Drug and Alcohol Dependence*, *129*(1-2), 151-157. <https://doi.org/10.1016/j.drugalcdep.2012.10.005>

Kelly, J. F., Urbanoski, K. A., Hoeppner, B. B., & Slaymaker, V. (2011). Facilitating comprehensive assessment of 12-step experiences: A Multidimensional Measure of Mutual-Help Activity. *Alcoholism Treatment Quarterly*, *29*(3), 181-203. <Go to ISI>://MEDLINE:22081741

Kessler, R. C., Barker, P. R., Colpe, L. J., Epstein, J. F., Gfroerer, J. C., Hiripi, E., Howes, M. J., Normand, S. L. T., Manderscheid, R. W., Walters, E. E., & Zaslavsky, A. M. (2003). Screening for serious mental illness in the general population [Article]. *Archives of General Psychiatry*, *60*(2), 184-189. <https://doi.org/10.1001/archpsyc.60.2.184>

Laudet, A. B., Harris, K., Kimball, T., Winters, K. C., & Moberg, D. P. (2015). Characteristics of Students Participating in Collegiate Recovery Programs: A National Survey [Article]. *Journal of Substance Abuse Treatment*, *51*, 38-46. <https://doi.org/10.1016/j.jsat.2014.11.004>

Litt, M. D., Kadden, R. M., Cooney, N. L., & Kabela, E. (2003). Coping skills and treatment outcomes in cognitive-behavioral and interactional group therapy for alcoholism [Article]. *Journal of Consulting and Clinical Psychology*, *71*(1), 118-128. <https://doi.org/10.1037/0022-006x.71.1.118>

Miller, W. R., & Delboca, F. K. (1994). Measurement of Drinking Behavior Using the Form-90 Family of Instruments [Article]. *Journal of Studies on Alcohol*, 112-118. <https://doi.org/10.15288/jsas.1994.s12.112>

Miller, W. R., Tonigan, J. S., & Longabaugh, R. (1995). The Drinker Inventory of Consequences (DrInC): An instrument for assessing adverse consequences of alcohol abuse. Test manual. In M. E. Mattson & L. A. Marshall (Eds.), *Project MATCH Monograph Series* (Vol. 4). National Institute on Alcohol Abuse and Alcoholism.

National Cancer Institute. (2009). *Tobacco Use Supplement to the Current Population Survey: Reports and publications using the TUS-CPS*. <http://riskfactor.cancer.gov/studies/tus-cps/publications.html>

National Institutes of Health, & U.S. Food and Drug Administration. (2013). Population Assessment of Tobacco and Health (PATH) Study: Final Adult Baseline (Wave 1) Questionnaire. In. Bethesda, MD: National Institutes of Health.

Prochaska, J. O., Velicer, W. F., Diclemente, C. C., & Fava, J. (1988). Measuring Processes of Change - Applications to the Cessation of Smoking [Article]. *Journal of Consulting and Clinical Psychology*, *56*(4), 520-528. <https://doi.org/10.1037/0022-006x.56.4.520>

Prorok, P. C., Andriole, G. L., Bresalier, R. S., Buys, S. S., Chia, D., Crawford, E. D., Fogel, R., Gelmann, E. P., Gilbert, F., Hasson, M. A., Hayes, R. B., Johnson, C. C., Mandel, J. S., Oberman, A., O'Brien, B., Oken, M. M., Rafla, S., Reding, D., Rutt, W., . . . Team, P. P. (2000). Design of the Prostate, Lung, Colorectal and Ovarian (PLCO) Cancer Screening Trial [Review]. *Controlled Clinical Trials*, *21*(6), 273S-309S. <https://doi.org/10.1016/s0197-2456(00)00098-2>

Robins, R. W., Hendin, H. M., & Trzesniewski, K. H. (2001). Measuring global self-esteem: Construct validation of a single-item measure and the Rosenberg self-esteem scale [Article]. *Personality and Social Psychology Bulletin*, *27*(2), 151-161. <https://doi.org/10.1177/0146167201272002>

Schmidt, S., Muhlan, H., & Power, M. (2006). The EUROHIS-QOL 8-item index: psychometric results of a cross-cultural field study [Article]. *European Journal of Public Health*, *16*(4), 420-428. <https://doi.org/10.1093/eurpub/cki155>

Underwood, L. G., & Teresi, J. A. (2002). The daily spiritual experience scale: Development, theoretical description, reliability, exploratory factor analysis, and preliminary construct validity using health-related data [Article]. *Annals of Behavioral Medicine*, *24*(1), 22-33. <https://doi.org/10.1207/s15324796abm2401_04>

Vilsaint, C. L., Kelly, J. F., Bergman, B. G., Groshkova, T., Best, D., & White, W. (2017). Development and validation of a Brief Assessment of Recovery Capital (BARC-10) for alcohol and drug use disorder [Article]. *Drug and Alcohol Dependence*, *177*, 71-76. <https://doi.org/10.1016/j.drugalcdep.2017.03.022>

Warttig, S. L., Forshaw, M. J., South, J., & White, A. K. (2013). New, normative, English-sample data for the Short Form Perceived Stress Scale (PSS-4) [Article]. *Journal of Health Psychology*, *18*(12), 1617-1628. <https://doi.org/10.1177/1359105313508346>

Wewers, M. E., & Lowe, N. K. (1990). A Critical Review of Visual Analog Scales in the Measurement of Clinical Phenomena [Review]. *Research in Nursing & Health*, *13*(4), 227-236. <https://doi.org/10.1002/nur.4770130405>

Zywiak, W. H., Neighbors, C. J., Martin, R. A., Johnson, J. E., Eaton, C. A., & Rohsenow, D. J. (2009). The Important People Drug and Alcohol interview: Psychometric properties, predictive validity, and implications for treatment [Article]. *Journal of Substance Abuse Treatment*, *36*(3), 321-330. <https://doi.org/10.1016/j.jsat.2008.08.001>
